# Critical roles of the Cu_B_ site in efficient proton pumping as revealed by crystal structures of mammalian cytochrome *c* oxidase catalytic intermediates

**DOI:** 10.1016/j.jbc.2021.100967

**Published:** 2021-07-15

**Authors:** Atsuhiro Shimada, Fumiyoshi Hara, Kyoko Shinzawa-Itoh, Nobuko Kanehisa, Eiki Yamashita, Kazumasa Muramoto, Tomitake Tsukihara, Shinya Yoshikawa

**Affiliations:** 1 Picobiology Institute, Graduate School of Life Science, University of Hyogo, kamigori, Akoh, Hyogo, Japan; 2 Department of Life Science, Graduate School of Life Science, University of Hyogo, kamigori, Akoh, Hyogo, Japan; 3 Department of Applied Chemistry, Graduate School of Engineering, Osaka University, Suita, Osaka, Japan; 4 Institute for Protein Research, Osaka University, Suita, Osaka, Japan

**Keywords:** catalytic intermediate, enzyme mechanism, metalloenzyme, cytochrome *c* oxidase (Complex IV), proton pump, X–ray crystallography, mitochondrial membrane potential, bioenergetics, heme, copper, CcO, cytochrome *c* oxidase, Cu_A_ and Cu_B_, low- and high-potential copper sites, DM, density modification, Fe_*a*_ and Fe_*a*3_, iron ions of heme *a* and heme *a*_3_, heme *a* and heme *a*_3_, low- and high-spin heme A molecules, IO10, IO20, and IO80, X-ray diffraction data sets, MR, molecular replacement, NCS, noncrystallographic symmetry, PDB, Protein Data Bank

## Abstract

Mammalian cytochrome *c* oxidase (CcO) reduces O_2_ to water in a bimetallic site including Fe_*a*3_ and Cu_B_ giving intermediate molecules, termed A-, P-, F-, O-, E-, and R-forms. From the P-form on, each reaction step is driven by single-electron donations from cytochrome *c* coupled with the pumping of a single proton through the H-pathway, a proton-conducting pathway composed of a hydrogen-bond network and a water channel. The proton-gradient formed is utilized for ATP production by F-ATPase. For elucidation of the proton pumping mechanism, crystal structural determination of these intermediate forms is necessary. Here we report X-ray crystallographic analysis at ∼1.8 Å resolution of fully reduced CcO crystals treated with O_2_ for three different time periods. Our disentanglement of intermediate forms from crystals that were composed of multiple forms determined that these three crystallographic data sets contained ∼45% of the O-form structure, ∼45% of the E-form structure, and ∼20% of an oxymyoglobin-type structure consistent with the A-form, respectively. The O- and E-forms exhibit an unusually long Cu_B_^2+^-OH^−^ distance and Cu_B_^1+^-H_2_O structure keeping Fe_*a*3_^3+^-OH^−^ state, respectively, suggesting that the O- and E-forms have high electron affinities that cause the O→E and E→R transitions to be essentially irreversible and thus enable tightly coupled proton pumping. The water channel of the H-pathway is closed in the O- and E-forms and partially open in the R-form. These structures, together with those of the recently reported P- and F-forms, indicate that closure of the H-pathway water channel avoids back-leaking of protons for facilitating the effective proton pumping.

Mammalian mitochondrial cytochrome *c* oxidase (CcO), the terminal oxidase of aerobic respiration, reduces molecular oxygen (O_2_) to water, coupled to proton pumping, providing charge separation (membrane potential) and proton gradient across the inner mitochondrial membrane for ATP production by F_o_F_1_ATP synthase (1, 2). The O_2_ reduction site of the enzyme contains two redox-active metal sites, Fe_*a*3_ and Cu_B_. Electrons for the O_2_ reduction are transferred from cytochrome *c* on the positive side (P-side) phase of the inner mitochondrial membrane (inter cristae side) to the O_2_ reduction site *via* the two low-potential metal sites, Cu_A_ and Fe_*a*_. Protons utilized during the reduction of O_2_ to water are transferred from the negative side (N-side) of the inner mitochondrial membrane (matrix side) *via* two proton-conducting pathways, termed D pathway and K pathway, to the O_2_ reduction site (Fig. 1*A*) (1, 2). Six catalytic intermediate forms, as described in Figure 1*B*, have been identified (1, 2). The reduced form, in which both Fe_*a*3_ and Cu_B_ are in the reduced state, termed the R-form (Fe_*a*3_^2+^, Cu_B_^1+^, and Tyr^244^-OH), traps O_2_ to provide the O_2_-bound A-form (Fe_*a*3_^2+^-O_2_, Cu_B_^1+^, and Tyr^244^-OH). The A-form relaxes into the P-form, designated as (Fe_*a*3_^4+^ = O^2−^, Cu_B_^2+^-OH^−^, and Tyr^244^-O•), where Tyr^244^-O• denotes the neutral radical of Tyr^244^. In this A- to P-form transition, three electrons from Fe_*a*3_^2+^ and Cu_B_^1+^ and one proton and one electron from Tyr^244^-OH are transferred to the bound O_2_ resulting in O^2−^ and OH^−^. Sequential donation of four electrons from cytochrome *c* to the O_2_ reduction site, including Fe_*a*3_ and Cu_B_, regenerates the R-form *via* three intermediate forms, F (Fe_*a*3_^4+^ = O^2−^, Cu_B_^2+^-OH^−^, and Tyr^244^-OH), O (Fe_*a*3_^3+^-OH^−^, Cu_B_^2+^-OH^−^, and Tyr^244^-OH), and E (Fe_*a*3_^3+^-OH^−^, Cu_B_^1+^-H_2_O, and Tyr^244^-OH) (Fig. 1*B*). Each electron transfer from cytochrome *c* to the O_2_ reduction site is coupled with the pumping of one proton equivalent from the N-side to the P-side (Fig. 1*B*) (1, 2, 3). On the basis of high-resolution X-ray structures and mutational studies of the proton pumping function of bovine CcO, it was proposed that a proton-conducting H-pathway that interacts with the periphery of heme *a* executes proton pumping in mammalian CcO, (1, 4, 5, 6). An alternative proton-pumping mechanism *via* the D-pathway has been proposed based mainly on mutational analyses of bacterial and yeast CcOs (1, 2, 7, 8). The simplest interpretation of this discrepancy could be evolutional diversity in the proton-pumping mechanism (1, 9).

**Figure 1:**
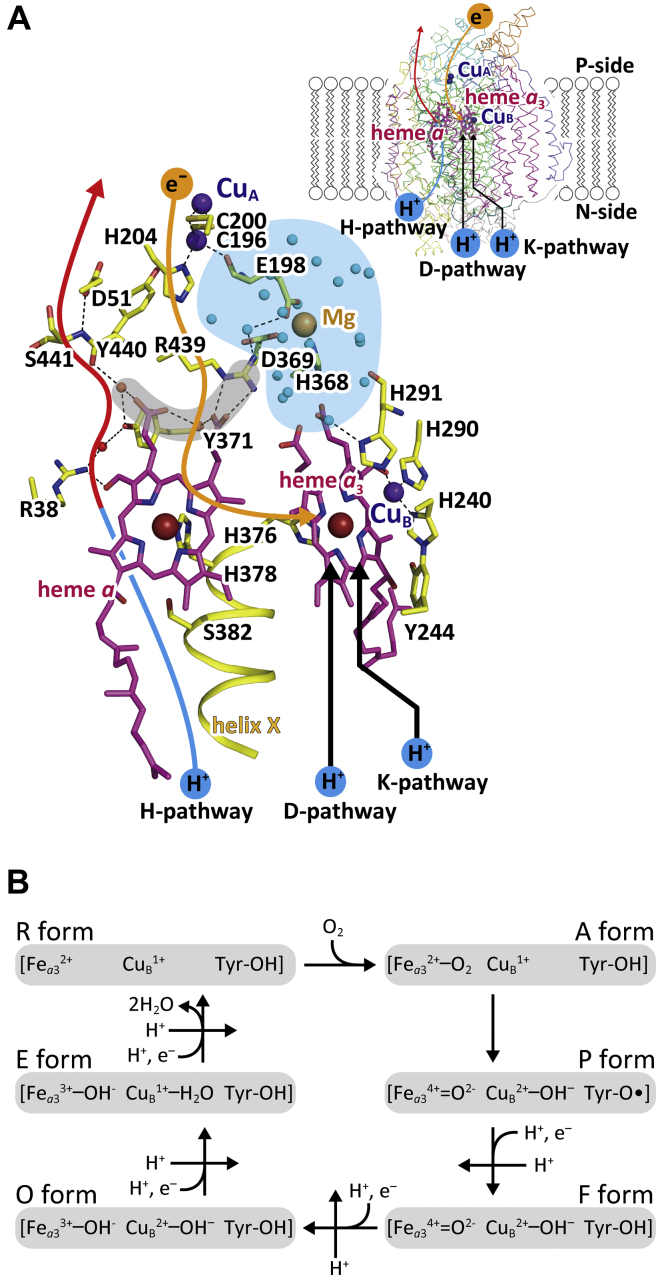
**X-ray structure of the active sites and a schematic representation of the catalytic cycle of bovine heart CcO.***A*, X-ray structure of the active sites. Metal sites are indicated by *brown*, *violet*, and *beige spheres* for iron, copper, and magnesium ions, respectively. Porphyrins of heme *a* and heme *a*_3_ are represented by the *magenta stick* models as labeled. Within the stick models of the amino acid residues, *dark blue*, *red*, and *yellow* portions are nitrogen, oxygen, and carbon, respectively. A *beige arrow* indicates the location of the electron transfer passage, while two *black arrows* indicate those for protons for producing water molecules. The hydrogen-bond network and the water channel of the H-pathway are indicated by the *red* and *blue* portions of the *leftmost curved arrow*, respectively. The Mg/H_2_O cluster (the *blue area*) is attached to the hydrogen-bond network of the H-pathway *via* a short hydrogen-bond network (the *gray area*). *Small blue spheres* in the Mg/H_2_O cluster mark the positions of water molecules, while *small beige spheres* mark the other water molecule positions. The formyl group and one of the propionate groups of heme *a* are hydrogen-bonded with Arg^38^ and a fixed water molecule in the hydrogen-bond network of the H-pathway. The *inset* shows the overall locations of the redox-active metal sites and pathways for transportation of electrons and protons within the CcO structure, indicated by Cα-backbone traces. This figure was prepared from the X-ray diffraction data of PDB 5B1A. *B*, a schematic representation of the structural changes in the O_2_ reduction site of CcO; Fe_*a*3_ and Cu_B_ are the iron and copper ions; Tyr-OH and Tyr-O• denote Tyr^244^ located in its protonated and neutral radical states, respectively. The six intermediate forms in the catalytic cycle are designated as A-to R-forms. The reaction steps coupled with proton pumping are indicated by *straight arrows* marked with “H^+^”. This figure is a slightly modified version of the previous paper (23).

For effective energy transduction in a redox-coupled proton-pumping system, the electron transfer must be essentially irreversible. Thus, the electron acceptor of the system should have a high electron affinity toward the electron donor of the system. In the case of CcO, the neutral radical state of Tyr^244^ (Tyr^244^-O•) and the ferryl state of Fe_*a*3_ (Fe_*a*3_^4+^ = O^2−^), detected by EPR and resonance Raman analyses, have been proposed to be those high electron affinity sites that provide essentially irreversible P→F and F→O transitions, respectively (10, 11, 12). While high electron affinity of the Cu_B_ site in the E state has been put forward based on evidence from absorption spectroscopy and electrometric analyses in hemes *a* and *a*_3_ during the O→E transition, direct structural evidence for the irreversible O→E and E→R transitions is missing (1, 13). Moreover, the essential colorlessness of the Cu_B_ site complicates examination of its configuration and functions in the O-form and E-form by spectroscopic means, thus making structural analyses by high-resolution X-ray crystallography for these reaction intermediates to be essential.

The fully oxidized CcO preparation purified under aerobic condition is designated to be in the resting oxidized form. In contrast to the O-form, it does not function as a proton pump (1, 14). Under aerobic conditions in the absence of an electron donor system, the O-form relaxes slowly (within a timescale of seconds to minutes) to the resting oxidized form (13, 14). High-resolution X-ray structural analysis indicates that the O_2_-reduction site of the resting oxidized form of bovine CcO is in a peroxide-bound form (15). This structure-based assignment has been confirmed by reductive titration (16) and resonance Raman analysis (17). However, X-ray structural analysis of the O-form is still missing.

The timescale of the catalytic cycle of CcO in solution (shorter than a few msec) (18) strongly suggests the necessity of time-resolved X-ray structural analysis by XFEL using caged O_2_ in order for the X-ray structural analysis of CcO intermediate forms during catalytic turnover. A recent report for application of this method to the bovine CcO crystal system indicates that the resolution of the electron density maps is not sufficient for identification of the ligand-binding structure in the O_2_ reduction site. For example, at the resolution, crystallographic discrimination between Fe^3+^-OH^−^ and Fe^4+^ = O^2−^ is not feasible (19). Significant improvements in CcO sample preparation method seem critical for successful application of this type of XFEL analysis.

Recent structural studies of various functional proteins by XFEL indicate that chemical processes in the proteins are often greatly slowed down or even blocked at intermediate states by crystal packing (20, 21). Therefore, for the visualization of the elusive intermediate forms of mammalian cytochrome *c* oxidase reaction cycle, we turned to synchrotron radiation X-ray crystallography to examine the structural changes induced by the oxidation of fully reduced CcO crystals caused by O_2_ exposure at three different time intervals. Each of the three individual data sets contained multiple structures. The three data sets included the O- and E-form structures at ∼45% occupancy and an oxymyoglobin-type structure at ∼20% occupancy, respectively. The O- and E-form structures suggested essentially irreversible O→E and E→R transitions.

## Results

### Absorption spectral changes induced by exposure of the fully reduced CcO crystals to excess amounts of O_2_

**Figure 2:**
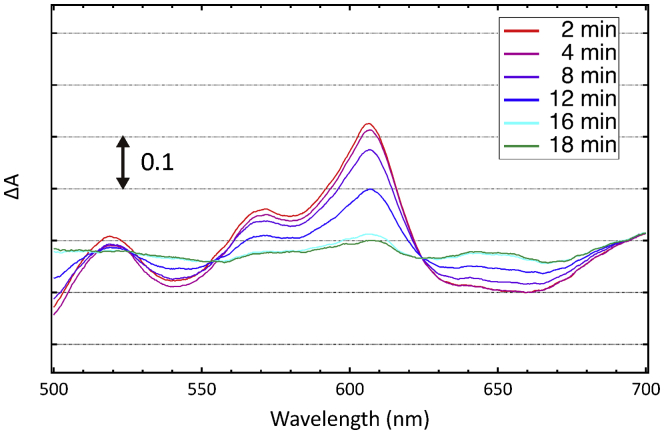
**Typical absorption spectral changes during oxidation of a fully reduced CcO crystal at 4 °C.** Each trace is a difference spectrum against the spectrum of the resting oxidized form crystal taken before reduction of the crystal. The oxidation of the reduced CcO was initiated by exchanging the medium containing the reducing system with an O_2_-saturated medium.

Figure 2 shows typical absorption spectral changes observed for the fully reduced bovine CcO crystals upon exposure to excess amounts of O_2_ at 4 °C in the α-band region where the contribution of reduced heme *a* is the highest. Significant absorption spectral changes due to CcO oxidation were detectable upon exposure to O_2_ after 20 min. Since in solution fully reduced CcO is completely oxidized to the O state by O_2_ within several milliseconds (18), oxidation of crystalline CcO in our experimental setup is likely rate-limited by the diffusion of O_2_ into the crystals. Thus the rate of spectral changes would also depend on the size of the crystal. To circumvent this problem, crystals with very similar sizes were used for both spectroscopic measurements and X-ray diffraction experiments. The observed fairly reproducible rate of spectral changes in the crystals suggests sufficient similarity for the crystal state in the present X-ray diffraction measurements.

The difference spectrum of reduced crystals after 18 min exposure to O_2_ against that of the resting oxidized form was not flat (Fig. 2), suggesting that the O-form has not completely been transformed into the resting oxidized form. This is in contrast to the case in solution where the transition from the O-form to the resting oxidized form is completed within a few minutes (13, 14). A dull peak around 607 nm detectable in the difference spectrum at 18 min in Figure 2 was gradually weakened in the following 60 min, suggesting that intramolecular electron transfer slowed down significantly due to crystal packing.

### X-ray structural analyses

X-ray diffraction experiments were carried out with dithionite-reduced crystals after exposure to excess O_2_ for approximately 10 min, 20 min, and 80 min, using 10, 6, and 12 crystals, respectively (Table 1). These three X-ray diffraction data sets are designated as IO10, IO20, and IO80. The asymmetric unit of each of the crystals used in this study contains two monomers of CcO, termed A and B, with each monomer comprising 13 different protein subunits.

**Table 1:** Statistics of intensity data collection

Crystal	IO10	IO20	IO80
O_2_ exposure periods (min)	5–10	20	45–90
Beam lines		BL44XU	
Beam sizes (μm)		50 (v) × 30 (h)	
Space groups		*P*2_1_2_1_2_1_	
Cell constants (Å)			
*A*	181.77	181.44	181.67
*B*	203.30	203.09	203.41
*C*	177.57	177.79	177.67
Number of crystals	10	6	12
Number of images	648	560	720
Resolution (Å)	137.0–1.74 (1.76–1.74)	137.0–1.84 (1.86–1.84)	137.0–1.76 (1.78–1.76)
Observed reflections	5,752,786	4,113,495	6,825,118
Independent reflection	663,784 (16,459)	561,788 (13,951)	644,860 (15,945)
Averaged redundancy	8.7 (6.7)	7.3 (5.8)	10.6 (7.8)
Completeness (%)	99.8 (99.9)	99.8 (99.8)	99.9 (99.9)
*R*_merge_	11.2 (>100.0)	9.60 (89.6)	10.2 (91.5)
*R*_pim_	3.3 (36.2)	3.3 (40.0)	2.9 (34.7)
*I*/σ(*I*)	30.6 (1.5)	31.7 (4.4)	37.3 (3.9)

Numbers in parentheses are given for the highest resolution shells. F_o_ data of the fully reduced form in the original PDB file (PDB ID code 5B1B) were used in the present study, whose statistics are given in Table 2 of the previous paper (9).

In this paper, the fully reduced form denotes a CcO form in which all of the redox metal sites are in the reduced state, (*i.e.*, Fe_*a*_^2+^, Cu_A_^1+^, Fe_*a*3_^2+^, and Cu_B_^1+^), while a form having Fe_*a*3_^2+^ and Cu_B_^1+^ regardless of the oxidation states of Fe_*a*_ and Cu_A_ is designated as the R-form. The structures detectable in the resting oxidized (PDB ID codes 5B1A and 3WG7) and fully reduced forms (PDB ID code 5B1B), both previously determined with singular structure models, are designated as the oxidized-type and reduced-type structures, respectively.

Structure determination procedure for the above three data sets, IO10, IO20, and IO80, employed here, consists of three steps: the first step is the structure determination procedure of a model with a singular structure; the multiple structures were identified for several parts of the molecule in the second step; and the structure of dioxygen reduction center was determined in the third step. The structure of bovine CcO determined previously at 1.6 Å resolution in the fully reduced form (PDB ID code 5B1B) was re-refined by this procedure using the *F*_*o*_ data obtainable from the PDB data set to improve its quality.

### The first step: Structural determination of a model with a singular structure

**Figure 3:**
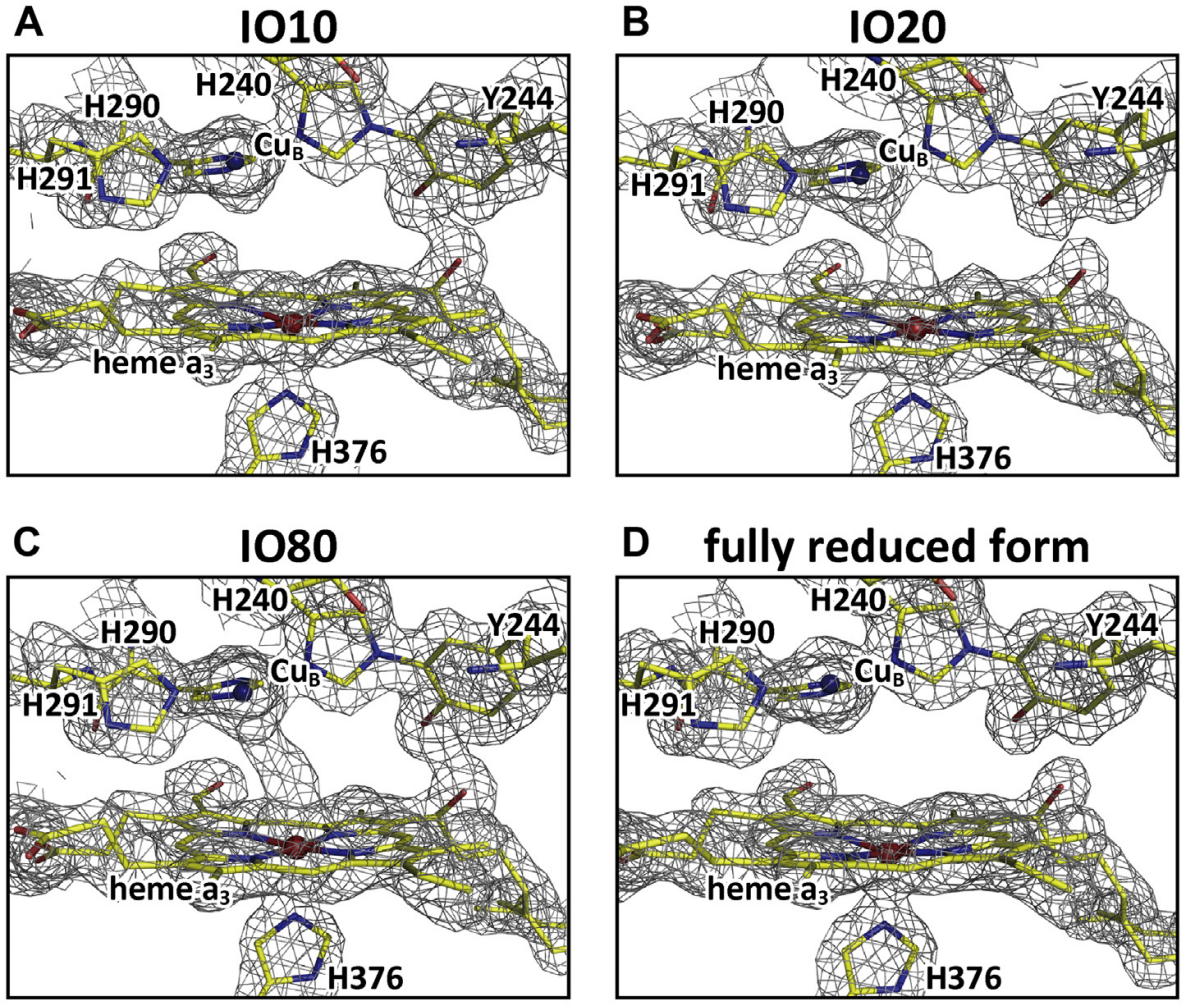
**The MR/DM electron density maps of the O**_**2**_**reduction sites, obtained from the four data sets of monomer A.***A*–*D*, from data sets, IO10, IO20, IO80, and the fully reduced form (PDB code 5B1B), respectively. Structures of proteins and hemes are drawn as stick models, and the Fe atoms in heme *a*_3_ and Cu atoms in Cu_B_ site are indicated by *beige* and *blue spheres*, respectively. Resolutions of the MR/DM maps were 1.74 Å, 1.84 Å, 1.76 Å, and 1.60 Å, respectively. Electron density cages of the MR/DM maps were drawn at 1.5 σ.

A detailed description for the first step is given in Experimental procedures. The singular structure model obtained for the IO10 data set is almost identical to the structures of the previously reported fully reduced form (PDB ID code 5B1B) (9), while the singular structures for the IO20 and IO80 data sets are almost identical to that of the previously reported resting oxidized form (PDB ID codes 5B1A and 3WG7) (9, 22). The MR/DM electron density maps of the O_2_ reduction sites, obtained from the three new data sets IO10, IO20, and IO80, together with that of the fully reduced form, are given in Figure 3.

### The second step: Determination of multiple structures.

**Figure 4:**
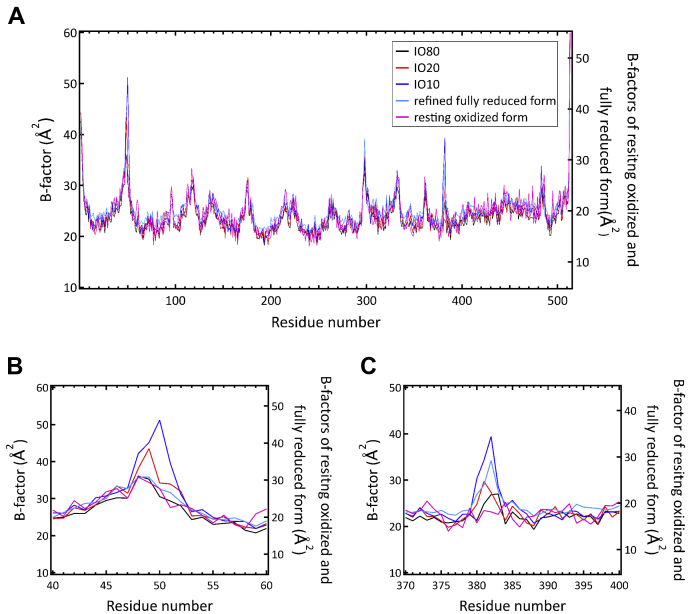
***B*-factors of the main chain portions (-NH-Cα-CO-) of the residues in the amino acid sequences of subunit I of the singular structures derived from IO10, IO20, IO80, the refined fully reduced form, and the resting-oxidized form (PDB code****3WG7****).***A*, *B*-factor values in Å^2^ are plotted against residue numbers of subunit I for IO10 (*blue*), IO20 (*red*), IO80 (*black*), the refined fully reduced form (*cyan*), and the resting-oxidized form (*magenta*). *B*, B-factor distributions near residue 49. *C*, *B*-factor distributions near residue 383.

For detecting the coexistence of multiple structures, we plotted the *B*-factors of the main chain portions (-NH-Cα-CO-) of the residues in the amino acid sequences of subunit I of each singular structure determined from IO10, IO20, and IO80, together with those of the re-refined fully reduced form and the damage-free resting oxidized form (PDB ID code 3WG7) (22) as given in Figure 4*A*. The *B*-factor values given in Figure 4 were obtained using the main chain atoms of monomer A. Previously, it was shown that redox-coupled structural changes between the fully reduced and resting oxidized forms are detectable in the two regions between residues 48 and 55 including Asp^51^ and between residues 380 and 385 including Met^383^ (23). The *B*-factor distributions in these two regions, given in Figure 4, *B* and *C* respectively, indicate that *B*-factor values of the resting oxidized form are the lowest among those of the five structures shown. The significantly higher average *B*-factor values of the hydroxyfarnesyl ethyl group of heme *a*, compared with that of the rest of the heme *a* molecule, are detectable in the above singular structures except for the resting oxidized CcO. These high *B*-factor values could be induced either by the existence of an additional structure as a minor component or by high thermal motions.

If a minor component exists in an electron density map calculated as a singular structure, the *F*_o_*-F*_c_ map against the major component structure would provide positive and negative electron densities corresponding to the minor and major component structures, respectively. Thus, the *F*_*o*_*-F*_*c*_ map calculation should be a sensitive, though qualitative, method for identifying the coexistence of the minor component.

**Figure 5:**
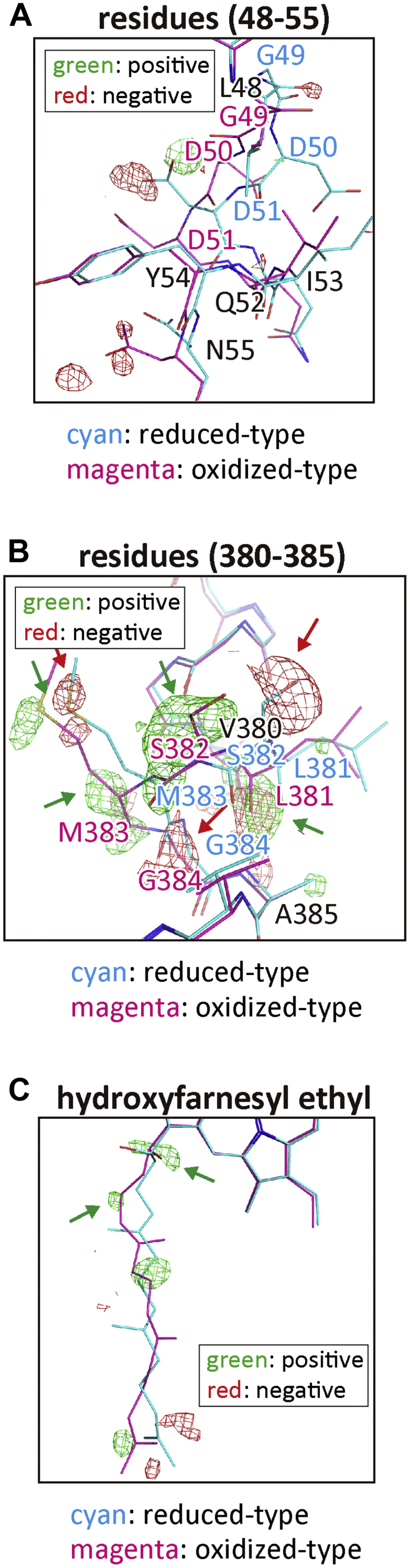
**An examination of coexistence of the minor component in the re-refined reduced form by *F*_o_-*F*_c_ map calculation.** The *F*_o_-*F*_c_ maps for the re-refined fully reduced form in the the following regions including residues 48 to 55 (*A*), residues 380 to 385 (*B*), and the hydroxyfarnesyl ethyl group of heme *a* (*C*), each giving positive (*green*) and negative (*red*) residual densities and the oxidized (*magenta*)- and reduced (*cyan*)-type structures. Electron density cages of the *F*_*o*_*-F*_*c*_ maps were drawn at 3.0 σ. In panel *B*, representative positive density cages on the oxidized-type model are indicated by *green arrows*, and representative negative density cages on the reduced-type model are indicated by *red arrows*. However, panel *A* does not show any representative electron density cage, and panel *C* provides two representative positive cages indicated by *green arrows*. This figure represents the results for monomer A.

If the oxidized-type structure is included as a minor component in the present re-refined fully reduced form structure, the *F*_o_*-F*_c_ map would provide negative and positive difference electron densities at the atomic positions of the reduced-type and oxidized-type structures, respectively. The *F*_o_*-F*_c_ maps of the regions between residues 48 and 55 and between residues 380 and 385 and of the hydroxyfarnesyl ethyl group of heme *a* were shown in Figure 5, *A*–*C*. In each *F*_o_*-F*_c_ map, the atomic models of the oxidized (PDB ID code 5B1A) and reduced (PDB ID code 5B1B) structures (magenta and cyan respectively) are superimposed.

The *F*_o_*-F*_c_ map for the region of residues 380 to 385 (Fig. 5*B*) exhibited several negative and positive densities at the positions corresponding to the atomic models of the reduced- and oxidized-type structures as marked by red and green arrows, respectively. The resulting map suggests coexistence of the oxidized-type structure as a minor component. In contrast the *F*_o_*-F*_c_ map for residues 48 to 55 shows no electron density cage at the atomic positions of either the reduced- or oxidized-type structures (Fig. 5*A*). Consequently, the high *B*-factor values in this region are likely caused by high thermal motion. In the hydroxyfarnesyl ethyl group region of heme *a*, two positive electron densities are detectable at the positions corresponding to positions in the oxidized-type structure (green arrows in Fig. 5*C*) indicating the presence of the oxidized-type structure as a minor component. To clarify the performance of the method employed in this study for the search of minor components in a given data set, three regions showing only negative or positive electron densities with the atomic models of the major or minor components are presented in Fig. S1.

Similar minor component searches were applied to the newly collected data sets IO10, IO20, and IO80 by examinations of *F*_o_*-F*_c_ maps for the three structural regions including residues 48 to 55, residues 380 to 385, and the heme *a* hydroxyfarnesyl ethyl group (Figs. S2–S4). Coexistence of the oxidized-type structures as minor components in all three structural regions of the electron density map obtained from the data set IO10 is detectable in the *F*_*o*_*-F*_*c*_ maps given in Fig. S2. The reverse situation was observed for the data sets IO20 and IO80 in which reduced-type structures are detectable as minor components in all three structural regions of the *F*_*o*_*-F*_*c*_ maps (Figs. S3 and S4). While these observations were made using monomer A, similar results were also obtained using monomer B.

**Figure 6:**
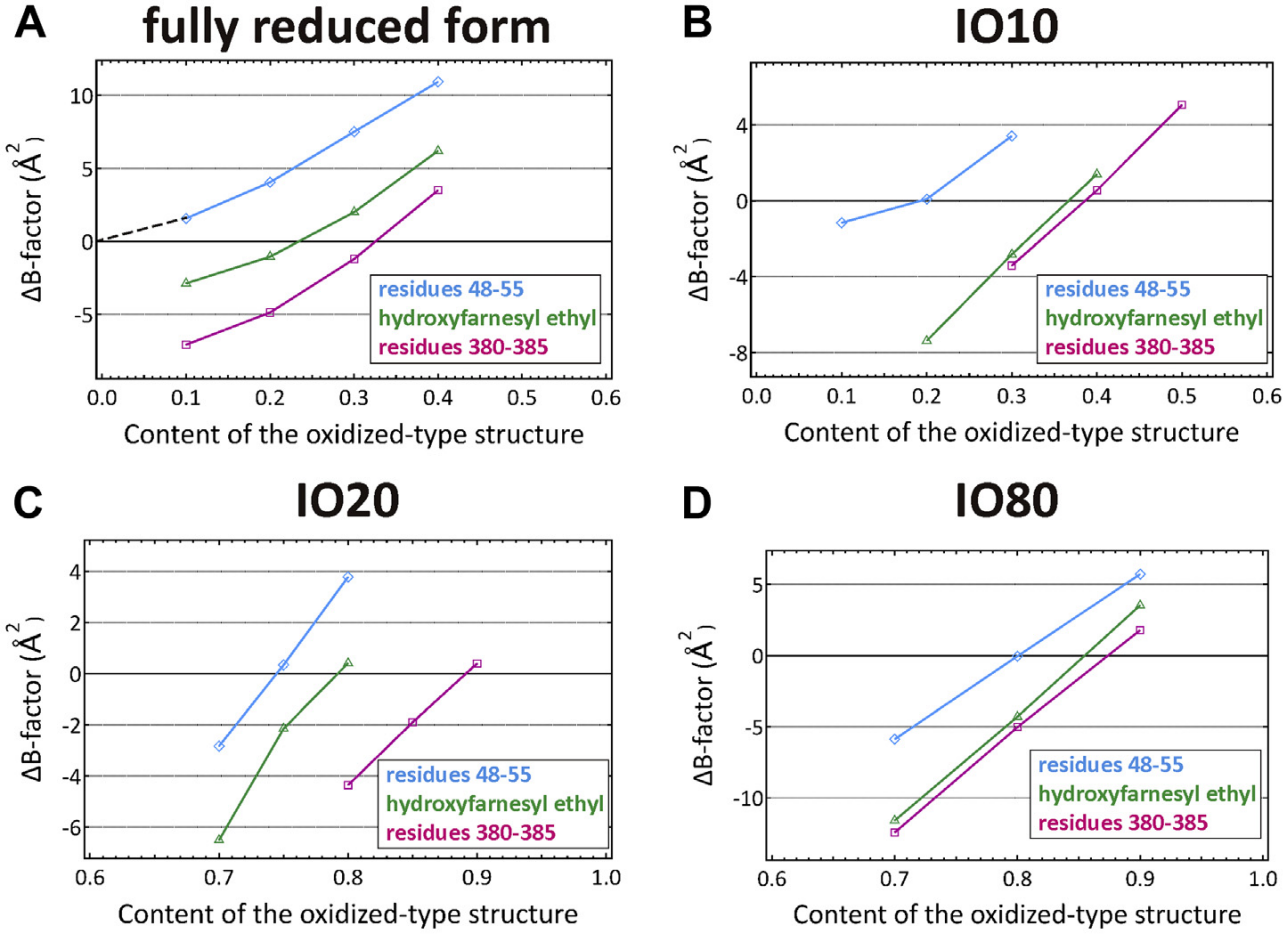
**Effect of content of the oxidized-type structure on the average *B*-factor values of the refined structures of both the oxidized- and reduced-type structures for the monomer A.** The effect is shown by the difference between the average *B*-factor values of the refined oxidized- and reduced-type structures (Δ*B*), defined as follows: Δ*B* = average *B*-factor value of the refined reduced-type structure – average *B*-factor value of the refined oxidized-type structure. The Δ*B* values, determined at various content of the reduced-type structure, are plotted against the content of the oxidized-type structure included, for the hydroxyfarnesyl ethyl group of heme *a* in *green*, and for residues 48 to 55 and 380 to 385, in *blue* and *red plots*, respectively. *A*–*D*, the Δ*B* plots for the fully reduced form, IO10, IO20, and IO80, respectively.

It is reasonable to assume that, in a multiple structure, all of the component structures have an identical average *B*-factor value. The contents of the minor and major components in a two-component structure can therefore be determined by searching the content ratio of the two components that yield an identical average *B*-factor for both component structures. Using this analytical approach, we estimated contents of the reduced-type and the oxidized-type structures for data sets of the fully reduced form (PDB ID code 5B1B), IO10, IO20, and IO80. *B*-factor differences between the reduced-type and oxidized-type structures (*ΔB*s) were plotted against the content of the oxidized-type structure in the regions of residues 48 to 55 and residues 380 to 385 of subunit I and the hydroxyfarnesyl ethyl group of heme *a* for monomer A (Fig. 6). The plots for 380 to 385 regions (red) and the hydroxyfarnesyl ethyl group (green) intersect the horizontal axis (the zero line), indicating coexistence of the oxidized-type and reduced-type structures in these regions. The blue plot in Figure 6*A* extrapolates to zero suggesting the absence of the oxidized-type structure, which is consistent with the *F*_o_*-F*_c_ map for the fully reduced form (Fig. 5*A*). Thus, the high *B*-factors in this region are due to high thermal motions. The same *B*-factor analysis was also performed for the data sets IO10, IO20, and IO80 indicating the coexistence of reduced and oxidized states (Fig. 6). This *B*-factor analysis was performed also for monomer B (Fig. S5). The contents of the oxidized-type structure estimated by these intersection points in the 48 to 55 region for the fully reduced form (PDB ID code 5B1B), IO10, IO20, and IO80, averaged for the two cytochrome *c* oxidase monomers of the crystallographic unit cell, are 0, 0.17, 0.72, and 0.79, respectively. And those for the region of residues 380 to 385 are 0.33, 0.42, 0.91, and 0.90, respectively. And those for the hydroxyl farnesylethyl group of heme *a* are 0.23, 0.37, 0.78, and 0.81, respectively.

This method of disentangling multiple structures had previously been applied successfully for the quantitative evaluation of the ratio of the reduced-type and the oxidized-type structures in H_2_O_2_-treated crystals of bovine CcO (23). (Absorption spectra of the crystals indicate that the oxidized-type structure is a 1:1 mixture of the F-form and the P-Form, while no significant X-ray structural difference was detectable between the two forms (23).)

### The third step: Structure determination of the O_2_ reduction site and final structural refinements

**Figure 7:**
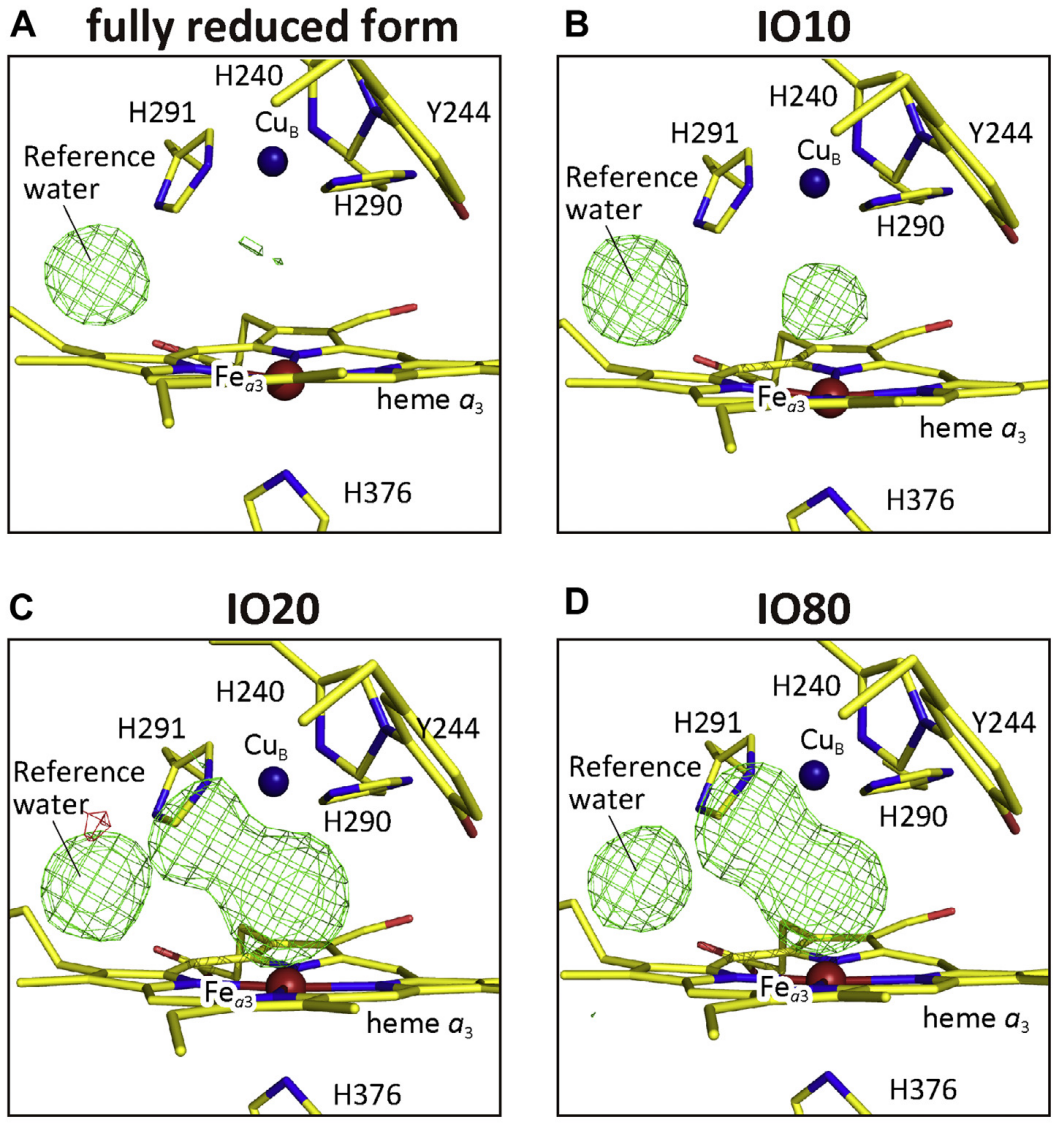
**Effect of O_2_ exposure periods on the electron density between Fe*_a_*_3_ and Cu_B_.***F*_o_-*F*_c_ maps of the O_2_ reduction site of the X-ray structures of the re-refined fully reduced form (*A*), IO10 (*B*), IO20 (*C*), and IO80 (*D*) in monomer A at 1.80 Å resolution. Refinement was performed on a structure in the absence of a ligand in the O_2_-reduction site and without the water molecule bridging the two propionate groups of heme *a*_3_, and the *F*_o_-*F*_c_ map was calculated. The electron density cages were drawn at 3.0 σ. Structures of proteins and hemes are drawn as *stick* models, and the Fe atoms in heme *a*_3_ and Cu atoms in Cu_B_ site are indicated by *red* and *dark blue spheres*, respectively.

Structures with multiple components, as determined in the second step, were refined and *F*_o_*-F*_c_ maps were calculated to inspect ligand structures in the O_2_ reduction site for monomer A. During the refinement, a water molecule bridging the two propionates of heme *a*_3_ was excluded for its use as a reference for peak height comparison (Fig. 7, *A*–*D*).

#### For the data set of the fully reduced form (PDB ID code 5B1B)

**Figure 8:**
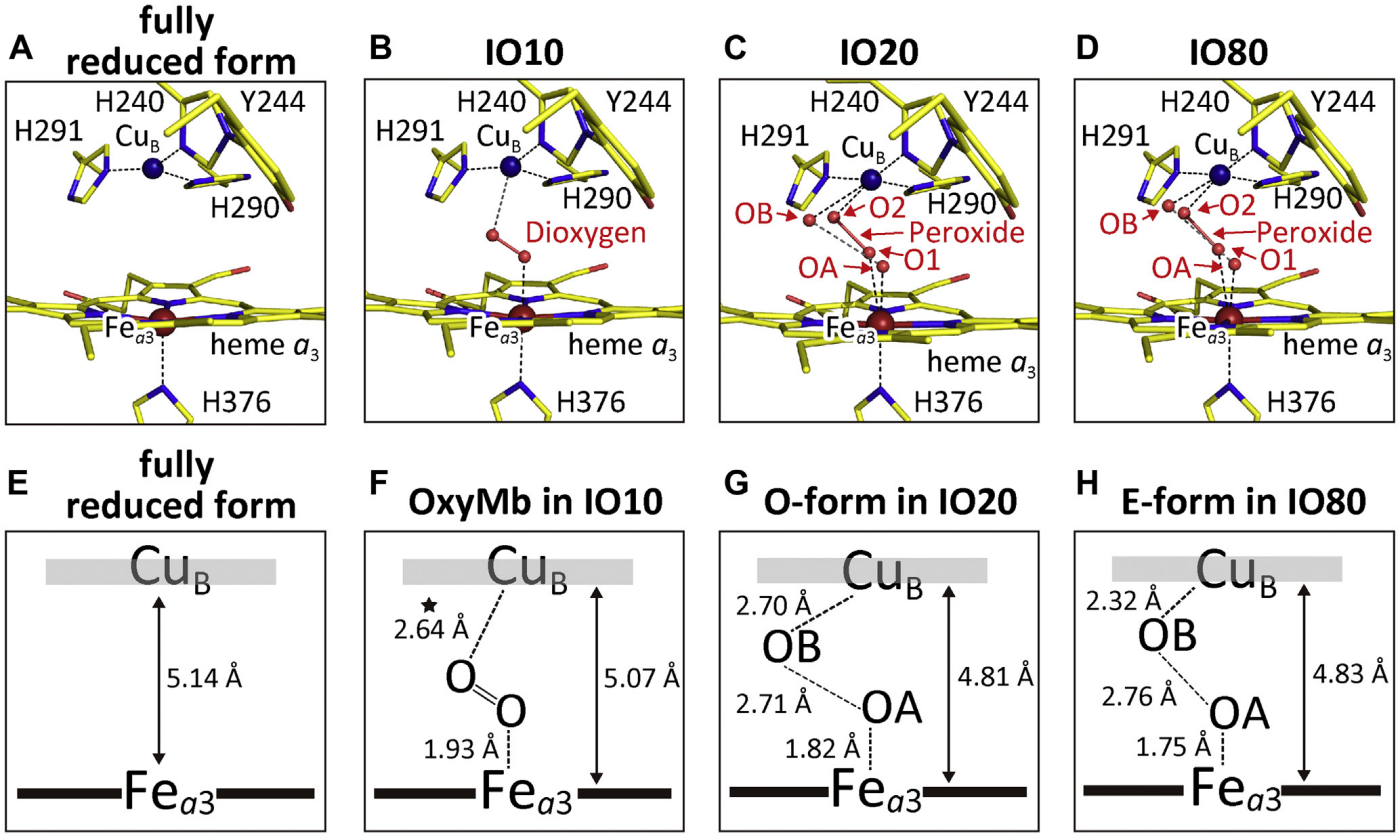
**Atomic models of the O**_**2**_**reduction site structures derived from the fully reduced form, IO10, IO20, and IO80 after final structural refinements.***A*, the final model of the refined fully reduced form. *B*, the final model from IO10, obtained by final refinement under a restraint condition of Fe-O = 1.93 Å for monomer A. The distance between Cu_B_ atom and the distal oxygen atom of the bound O_2_, averaged for the two monomers, is 2.64 Å. *C* and *D*, the final models, from IO20 and IO80, each composed of a peroxide-bound structure and a nonperoxide-bound structure, respectively, for monomer A. *E*, a schematic representation of the O_2_ reduction site structure of the re-refined fully reduced form given in *A*. *F*–*H*, schematic representations of the structures including nonperoxide ligands in the final models from IO10, IO20, and IO80, respectively. The distances given in *F*–*H* are average values between the two monomers. The reliability of the Cu_B_-O distance given in *F*, marked by an *asterisk*, is significantly lower than those given in *G* and *H*, because of the lower occupancy.

No significant electron density of *F*_o_*-F*_c_ maps in the ligand-binding space in the O_2_ reduction site is detectable. Thus, the structure was re-refined without any ligand around Fe_*a*3_ and Cu_B_. The resultant atomic model and a schematic representation of the O_2_ reduction site are shown in Figure 8, *A* and *E*, respectively.

Consistent to the minor component searches given above, the present X-ray structural analysis revealed that the oxidized-type structure existed in the 380 to 385 residue region and the hydroxy farnesyl ethyl group of the heme *a* as minor components (about 30%), while no significant oxidized-type structure was detectable in the 48 to 55 residue region, as shown in Table 2.

**Table 2:** Occupancies of oxidized-type structures

Data set	Region	Occupancy (monomer A, B)
IO10	Residues 48–55	0.18 (0.20, 0.15)
	Residues 380–385	0.40 (0.37, 0.42)
	Heme *a* side chain	0.35 (0.32, 0.38)
IO20	Residues 48–55	0.73 (0.75, 0.70)
	Residues 380–385	0.83 (0.83, 0.83)
	Heme *a* side chain	0.74 (0.72, 0.75)
IO80	Residues 48–55	0.86 (0.87, 0.85)
	Residues 380–385	0.90 (0.84, 0.95)
	Heme *a* side chain	0.82 (0.86, 0.78)
Fully reduced form	Residues 48–55	0.00 (0.00, 0.00)
	Residues 380–385	0.32 (0.32, 0.32)
	Heme *a* side chain	0.26 (0.28, 0.23)

Average values for monomers A and B in parentheses are given.

The absorption spectrum of crystalline CcO can be influenced significantly by the crystal packing, and the absorption spectral property of crystalline fully reduced CcO has not been well characterized. However, the absence of ligands at the O_2_ reduction site provides strong evidence for the completeness of CcO crystal reduction, as in the present case, since the absence of ligands at the O_2_ reduction site of the fully reduced CcO should be certain. Thus, the coexistence of the oxidized-type structure as a minor component is unlikely to be due to incomplete reduction of the CcO crystals. To our knowledge, the coexistence of the oxidized-type structure in the fully reduced form has never been reported thus far.

#### For the data set of IO10 containing an oxymyoglobin-type structure at ∼20%

The *F*_o_*-F*_c_ map of the IO10 model had a low but significant electron density with a peak height of 6 σ (Fig. 7*B*). The electron density in the Cu_B_ ligand site was not detectable at 3 σ level as shown in Figure 7*B* and the O_2_ ligation structure of oxymyoglobin (PDB ID code 1A6M) (24) superposes well on the electron density. Thus, the peak is assignable as an O_2_-bound form. The structure is designated as the oxymyoglobin (OxyMb)-type structure. The occupancy of the peak was estimated to be ∼0.15 by comparing its peak height with that of the reference water molecule. The structure was refined under a restraint condition of Fe-O = 1.93 Å, which is the distance between Fe_*a*3_ of CcO and the proximal oxygen atom of O_2_ of myoglobin superposed on the CcO molecule. The final atomic model given in Figure 8*B* indicates that the distance between the Cu_B_ atom and the distal oxygen atom of the bound O_2_ is 2.64 Å (Fig. 8*F*). The occupancies of both the proximal and distal oxygen atoms of the bound O_2_ in the final structure were 0.20 for both monomers (Table 3).

**Table 3:** Occupancies of the ligand-binding states

Data set	Nonperoxide-bound	Ligand-free (Fully reduced)	Peroxide-bound (Resting oxidized)
IO10	0.20 (OxyMb-type)	0.80	0.0
IO20	0.45 (O)	0.15	0.40
IO80	0.45 (E)	0.10	0.45

The small occupancy of the OxyMb-type structure (0.20) indicates that the location of the Cu_B_ atom in the model is essentially identical to that of the fully reduced form. X-ray structures of the fully reduced CcO derivatives of O_2_-model compounds, CO and NO, showed slightly longer (5.28 Å) and shorter (4.92 Å) Cu_B_^1+^-Fe_*a*3_^2+^ distances than that of the fully reduced-CcO (5.12 Å) (PDB ID codes 5X1F, 3AG3, and 5B1B, respectively). These results suggest that the Cu_B_^1+^-Fe_*a*3_^2+^ distance of the OxyMb-type structure (an O_2_-bound fully reduced CcO) is between 5.28 Å and 4.92 Å. In other words, it is unlikely that the Cu_B_^1+^-O_2_ distance the OxyMb-type structure is 0.2 Å shorter than 2.64 Å. The shortest possible distance (2.44 Å) is definitely longer than that between Fe_*a*3_ and the proximal oxygen of the bound O_2_ (1.93 Å). We tentatively propose that the Cu_B_^1+^-O_2_ distance of the OxyMb-type structure is 2.64 Å. A schematic representation of the structure of the A-form is shown in Figure 8*F*. Because of the low occupancy, the reliability of the calculated Cu_B_-O distance, 2.64 Å, given in Figure 8*F*, is not as high as those for the O-form and the E-form given in Figure 8, *G* and *H*.

The average occupancies of the oxidized-type structures for the two monomers in the 48 to 55 and 380 to 385 residue regions and the heme *a* side chain in the final structures obtained from IO10 data set are 0.18, 0.40, and 0.35, respectively (Table 2). The fully reduced form (*i.e.*, the ligand-free form) of 0.80 occupancy (Table 3) provides the oxidized type structures of 0.00, 0.26, and 0.21 for these three regions, respectively. Thus, the rest of the occupancies of the oxidized-type structures, 0.18, 0.14, and 0.14, respectively, should be due to the ligand-bound form (the OxyMb-type form). These occupancies are consistent with the occupancy of the OxyMb-type form, 0.20, within the experimental accuracy (Table 3). This indicates that the OxyMb-type form has the oxidized-type structure in these three regions. In other words, heme *a* is in the oxidized state and the water channel is closed. The occupancy of the oxidized heme *a*, 0.14, is consistent with the absorption spectral decrease in the α-band as given in Figure 2. Further arguments on the assignment of the OxyMb-type structure are given in Discussion.

#### For the data set of IO20 containing the O-form structure at ∼45%

The *F*_o_*-F*_c_ map of the IO20 model has two peaks between Fe_*a*3_ and Cu_B_ (Fig. 7*C*). Their peak heights were 0.95 and 0.53 of that of the reference water at the Fe_*a*3_ and Cu_B_ sites, respectively, in monomer A (Fig. 7*C*), and 0.89 and 0.57 in monomer B. The two peaks were separated from each other by 2.28 Å on average. Since these distances are too short for two nonbonding oxygen atoms, and too long for covalent bonds, we assigned these peaks to a mixed structure of a peroxide anion with an O-O distance of 1.55 Å and two nonbonding oxygen atoms (designated as OA and OB, existing near the Fe_*a*3_ and Cu_B_ sites, respectively). A peroxide group was located by superposing the damage-free structure of the resting oxidized form (PDB ID code 3WG7) (22) on the IO20 protein structure. Setting the occupancy of peroxide at 0.10 intervals from 0.30 to 0.70, *F*_o_*-F*_c_ maps were calculated to estimate the occupancy of peroxide as shown in Fig. S6. Inspecting residual positive and negative peaks at the peroxide site, we estimated the peroxide occupancy to have a range between 0.40 and 0.50. Two nonperoxide oxygen atoms (OA and OB) were located in the *F*_o_*-F*_c_ map. Fixing the peroxide position, we refined the structure without any restraint for the two nonperoxide oxygen atoms. Two oxygen atom sites were converged to their distances of 1.84 Å from Fe_*a*3_ (OA) and 2.72 Å from Cu_B_ (OB). Further refinements were performed under restraints of 1.84 Å for Fe_*a*3_-OA and 2.72 Å for Cu_B_-OB. Occupancies of peroxide and nonperoxide atoms were adjusted by examining the *F*_*o*_*-F*_*c*_ map and their *B*-factors for each refinement calculation. The occupancies of the protein moieties and the hydroxyfarnesyl ethyl group of heme *a* were readjusted, and the reference water molecules were included in the calculation at the final stage of the refinement. The above analysis was conducted also for monomer B.

The final atomic model obtained by these analyses is given in Figure 8*C*. The model includes a resting oxidized form structure (22) with the O-O (O1-O2) bond distance as 1.55 Å and Fe_*a*3_-O1 and Cu_B_-O2 distances as 2.22 Å and 2.24 Å, respectively. The two oxygen atoms provide an identical occupancy, 0.40, for both monomers. The other form includes two nonbonding (nonperoxide) oxygen atoms (OA and OB) located 1.82 Å and 2.70 Å apart from Fe_*a*3_ and Cu_B_, respectively, as illustrated in Figure 8*G*. These distances are averages between those of the two monomers. The occupancies of OA and OB were 0.50 and 0.40, respectively, in both monomers. The average, 0.45, is given in Table 3. The occupancies of the oxidized-type structures for the two protein regions and the hydroxyfarnesyl ethyl group of heme *a* are summarized in Table 2.

The Fe_*a*3_-OA distance, 1.82 Å, is significantly longer than that of Fe_*a*3_^4+^ = O^2−^, 1.70 Å, in the P- and F-forms (23), suggesting a Fe^3+^-OH^−^ structure. Thus the other form is assignable to the O-form. Furthermore, the Cu_B_-OB distance of 2.70 Å is longer than that in structures of the P-form and F-form with 2.11 Å. The OA-OB distance, 2.71 Å, in the other form suggests an ordinary hydrogen bond, in contrast to the low-barrier (short) hydrogen bond in the P- and F-forms, 2.54 Å (23). These locations of OA and OB also support the above assignment of the other form as the O-form. The Cu_B_-OB distance of the O-form, 2.70 Å, is unusually long as compared with those of normal Cu^2+^-OH^−^ coordination compounds (Table S1), suggesting a very weak negative charge influence of OH^−^ on the Cu_B_.

The above analyses indicate that CcO crystals used for the IO20 data set consisted CcO of which 45% were in the O state, 40% in the resting-oxidized state, and 15% in the fully reduced state (Table 3). Mixed structures due to the existence of the fully reduced form were identified in the three regions (the residues 48–55 and 380–385 and the OH group of heme *a* hydroxyfarnesyl ethyl group), as summarized in Table 2. The total (0.85) of occupancies of the O-form (0.45) and the resting-oxidized form (0.4) estimated by the electron density map of the O_2_ reduction site, as described above (Table 3), is consistent with each of the occupancies of the oxidized-type structures in these three regions (0.73. 0.83, and 0.74). Therefore, both the resting-oxidized form and the O-form have these three regions in the oxidized-type structure. In effect, the water channel is closed in the O-form.

In the O_2_ reduction site in the *F*_o_*-F*_c_ map of IO20 (Fig. S8*A*), a water molecule with a partial occupancy was located at a site close to Tyr^244^ in the *F*_*o*_*-F*_*c*_ map drawn at 3.0 σ. The water molecule hydrogen bonded to Tyr^244^ was at the same location of the interstitial water in the P- and F-forms as previously reported (23). The result suggests that the O-form also has the interstitial water. In contrast, no positive peak is detectable around the OH group of Tyr^244^ in *F*_o_*-F*_c_ maps of the present re-refined fully reduced form and the resting-oxidized form (9).

#### For the data set of IO80 containing the E-form structure at ∼45%

In the *F*_*o*_*-F*_*c*_ maps of the IO80 data set, two clear density peaks separated by 2.00 Å are discernable at the ligand coordination position of the oxygen reduction site located between Fe_*a*3_ and Cu_B_ (Fig. 7*D*). The peak height at the Fe_*a*3_ site is 1.02 and 0.96 relative to that of the reference water for monomers A and B, respectively. And those at the Cu_B_ site are 0.82 and 0.77. We assigned a mixed structure of a peroxide anion and two ligand oxygen atoms with the same procedure as in the analysis of the IO20 data set. The occupancy of peroxide was estimated to be ∼0.50 (Fig. S7), which was slightly higher than that of IO20. The nonperoxide oxygen atoms, OA and OB, were located at 1.76 Å from Fe_*a*3_ and at 2.30 Å from Cu_B_, respectively. These distances were applied to restraints in the further structure refinements. The refinements were conducted like those for the IO20 data set. The final atomic model obtained by these analyses is shown in Figure 8*D*. The structure of the nonperoxide form included in the final atomic model is schematically illustrated in Figure 8*H*. The atomic distances given in the figure are average values between the A and B monomer.

The refined atomic model of the O_2_ reduction site of IO80 shows that by increasing the O_2_-exposure time from 20 min to 80 min, the distance between Cu_B_ and OB was decreased clearly from 2.7 Å to 2.3 Å without any significant change in the other region in the O_2_ reduction site as given in Figure 8, *G* and *H*. The Cu_B_-OB distance change induced by elongation of the O_2_-exposure period is detectable by *F*_o_*-F*_o_ electron density analysis as given in the next section. This slow structural change in the Cu_B_ site is consistent to a kinetic finding that, upon O to E transition, Cu_B_^2+^ is selectively reduced (13). Thus it is reasonable to assign the structural difference between the nonperoxide-bound forms in IO20 and IO80 as the one due to the O to E transition. Further arguments for this assignment are given in Discussion. As in the case of IO20, IO80 showed that the E-form had the interstitial water hydrogen-bonded to Tyr^244^ as shown in Fig. S8*B*.

The occupancies of O1, O2, OA, and OB in the final structure are 0.45, 0.45, 0.50, and 0.40, respectively, in both monomers A and B. Thus, the CcO crystals, from which the IO80 data set was taken, consisted of 45% of the E-form, 45% of the resting oxidized form, and 10% of the fully reduced (ligand-free) form as given in Table 3. The occupancies of the oxidized- and reduced-type structures for the two protein regions and the hydroxyfarnesyl ethyl group of heme *a* in the final structure are summarized in Table 2.

The small amount of the ligand-free form (the fully reduced form) still existed in the IO80 structure and provided mixed structures in the protein moieties and the heme *a* hydroxyfarnesyl ethyl group (Table 2). The occupancy of the oxidized-type structure in the helix X region (residues 380–385), 0.90 (Table 2), is essentially identical to the total of the occupancies of the resting oxidized and E-forms, indicating that the water channel is closed in the E-form.

#### Changes in the ligand-binding structure of the Cu_B_ site upon O-form to E-form transition as visualized by the F_o_-F_o_ map

**Figure 9:**
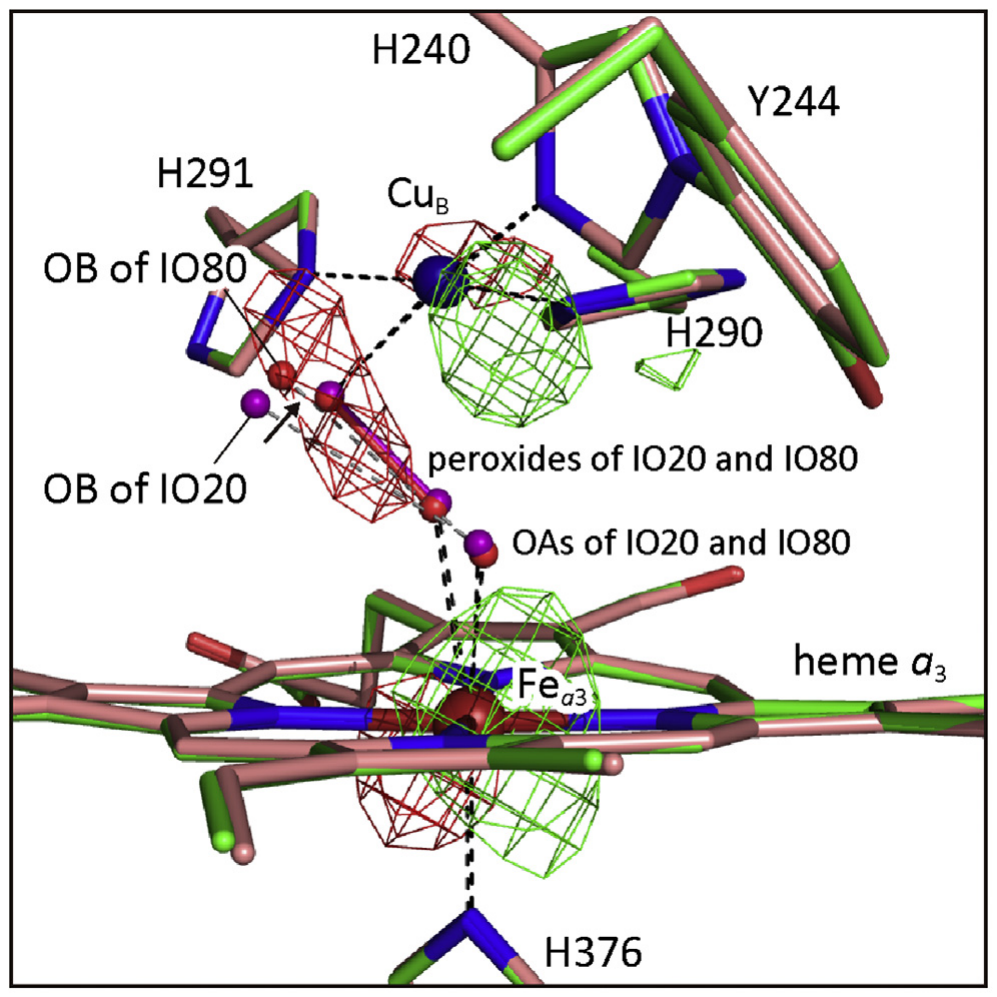
***F***_***o***_**(IO20)-*F***_***o***_**(IO80) electron density map calculated with IO80 phases in the O**_**2**_**reduction site.** Nitrogens and oxygens in heme *a*_3_ and amino acid residues are drawn by *dark blue* and *red sticks*. Carbons in IO80 and IO20 are *beige* and *green sticks*, respectively. *Small purple* and *red spheres* are nonbonding-oxygen atoms of IO80 and IO20, respectively. Two pairs of oxygen atoms linked with *purple* and *red sticks* denote the two peroxides shared by IO20 and IO80, respectively. Positive and negative densities are drawn at 4.0 σ by *green* and *red cages*, respectively. A pair of positive and negative peaks at each heavy atom site are consistent with a small (~0.06 Å) translational shift of CcO molecules upon transition from IO20 to IO80. A *black arrow* denotes the direction of migration of OB upon O→E transition.

The close similarities between the cell constants of IO80 and those of IO20 (Table 1) allow to detect the OB migration toward Cu_B_ upon the O- to E-form transition directly in the *Fo-Fo* electron density. The *F*_o_(IO20)-*F*_o_(IO80) electron density map was calculated with IO80 phases (Fig. 9). The locations of the nonbonding oxygen atoms located near the two metals are indicated by small purple and red spheres for IO80 and IO20, respectively. O-O bonds of the peroxides are marked by sticks with the same colors as those of the oxygen atoms in the figure. CcO molecules in IO80 are located at a position, translationally shifted by ∼0.06 Å from those in IO20. The small translational shift is indicated by a pair of positive and negative densities at each heavy atom site, drawn at 4.0 σ by green and red cages, respectively. Difference densities induced by the small translational shift are not significantly detectable for light atoms such as C, N, and O exhibiting only low electron densities. Significant negative density at a region including OB of IO80 and the peroxide is consistent with a shorter (∼0.4 Å) Cu_B_-OB distance of IO80 than that of IO20 and higher peroxide occupancy of IO80 than that of IO20, as described above, although positive electron density cages at the position of OB of IO20 are not clearly seen in this σ level. This *F*_o_(IO20)-*F*_o_(IO80) calculated with IO80 phases for monomer A indicates that the Cu_B_-OB distance in IO20 is significantly longer than that in IO80 in the map, confirming a shift of the oxygen atom (OB) toward the Cu_B_ site upon the O to E transition (Fig. 9). An essentially similar *F*_o_(IO20)-*F*_o_(IO80) map was also obtained for monomer B. Thus far, the lack of sufficient cell constant similarity between pairs of other reaction intermediates precluded the above described *F*_o_*-F*_o_ analysis.

The structural changes in the ligand-binding structure of the Fe_*a*3_ and Cu_B_ sites during the catalytic cycle, determined thus far, are summarized in the Movie S1 given in Supporting Information. The Oxy-Mb type structure, as a model of the A-form, is included in the Movie.

Some X-ray structural findings on the three proton conducting pathways, H, K, and D, of the newly determined intermediates are given in Supporting information, entitled as X-ray structural examination of flexibility of the critical residues of the substrate proton transfer pathways (Supporting Text 1), Gating of the substrate proton transfer pathways (Supporting Text 2), and A multiple structure of the hydroxyfarnesyl ethyl group of heme *a* (Supporting Text 3).

## Discussion

### Assignments of the X-ray structures to intermediate forms

Under the present experimental conditions, in which crystals of fully reduced mammalian CcO are oxidized by excess amounts of O_2_, the final product obtained by the O_2_-treatment is most likely to be the O-form in which all four redox-active metal sites are in the oxidized state (Cu_A_^2+^, Fe_*a*_^3+^, Fe_*a*3_^3+^, and Cu_B_^2+^), since further oxidation of the oxidized metals by O_2_ is energetically unfavorable. Therefore, the slow structural changes after the O-form formation are assignable to those induced by the formation of the E-form from the O-form by slow electron donations from the protein moiety of CcO. It has been reported that various amino acid residues in the protein moiety of CcO can act as redox-active metals (10, 11, 12). The structure of the resting oxidized form, which is a peroxide-bound oxidized form (9), provides evidence for the existence of such electron-donating amino acid residues in CcO, since these residues are highly likely to reduce O_2_ molecules, which diffuse to the O_2_ reduction site, to peroxides (15, 16).

The unusually long Cu_B_^2+^-OH^−^ distance in the O-form of 2.7 Å suggests that Cu_B_^2+^ has a very high redox potential, possibly because it is under weak influence of the negative charge from the bound OH^−^. Therefore, the structural change in the Cu_B_ site upon elongation of the O_2_-exposure time is reasonably assigned to the reduction of the Cu_B_ site upon the O-E transition. Upon this transition, the Cu_B_-OB distance was shortened to 2.3 Å, which is close to the usual coordination distance. If the negatively charged state in the ligand (OH^−^) is preserved upon the structural change, the shorter Cu_B_-OH^−^ distance could lower the electron affinity of Cu_B_. Then, Fe_*a*3_ would be in the partially reduced state in the E-form. However, no significant reduction in the Fe_*a*3_ was detectable. In fact, both the occupancy of OA and the Fe_*a*3_-OA distance (Fig. 8) were not influenced upon the O-E transition. Consistent with the X-ray structural finding, very small spectral changes were observed in the α-band region from 20 min to 80 min after initiation of O_2_ exposure of the dithionite-reduced CcO. This suggests that also in the E-form the redox potential of Cu_B_ is higher than that of Fe_*a*3_. The simplest interpretation for this experimental result would be protonation of the OH^−^ ligand of Cu_B_ (introduction of a positive charge to the ligand) upon reduction of Cu_B_^2+^.

The present assignment of the Cu_B_ site of the E-form as a Cu^1+^-H_2_O structure is consistent with X-ray structures of several organometallic compounds, each including a Cu^1+^-H_2_O coordination, in which the H_2_O ligation was determined by identification of the hydrogen atom positions (25, 26, 27). Further structural details reported thus far are summarized in Supporting Text 4 and Table S1. The structure, Cu_B_^1+^-H_2_O, has been previously proposed by Belevich *et al.* (13) based on spectrophotometric and electrometric analyses. Our present crystallographic findings support this proposal.

It has been shown that, in solution, the A-form shows a time-resolved resonance Raman band at 571 cm^−1^. The band position is essentially identical with those of the Fe^2+^-O_2_ stretching bands of oxymyoglobins and oxyhemoglobins (18). This suggests that the O_2_-binding structure is identical to those of oxymyoglobins and oxyhemoglobins and that the interaction between Cu_B_ and the distal oxygen atom of the O_2_ molecule at Fe_*a*3_ is essentially absent. This resonance Raman finding is consistent to the electron density of the bound ligand in IO10, given in the *F*_o_*-F*_c_ map of Figure 7*B*, showing that the bound ligand is in the oxymyoglobin-type structure and that the Cu_B_-O_2_ distance is significantly longer than the Fe_*a*3_-O_2_ distance. Thus, this electron density is reasonably assignable to that of the A-form, although its occupancy is only ∼20%. Considering the low occupancy, we designated the final atomic model (Fig. 8, *B* and *F*) obtained from the electron density (Fig. 7*B*) as the OxyMb-type structure, as described above.

Our findings suggest that the A-form, the lifetime of which is shorter than 0.3 ms in solution (18, 28, 29), is stabilized by the crystal packing, as in the case of various functional proteins (22, 23). High-resolution X-ray structural analysis for the A-form would be possible using the present CcO crystals, if the conditions for trapping the OxyMb-type structure in the crystals are improved.

### The roles of the Cu_B_ site in the catalytic mechanism of CcO

The present X-ray structural findings for the colorless copper site, Cu_B_, in the O- and E-forms, show that the Cu_B_ site creates high electron affinity structures for facilitating the essentially irreversible O→E and E→R transitions. A possible scenario is that the unusually long Cu_B_^2+^–OB distance in the O-form increases the electron affinity of Cu_B_^2+^ by decreasing the influence of the negatively charged OH^−^. Additionally, the proton availability to OB from the K-pathway (Fig. S9) *via* the interstitial water and OA could increase the electron affinity of the Cu_B_^2+^. Thus, it is reasonable to conclude that the essentially irreversible O→E transition is conveyed by the Cu_B_^2+^ site in the O-form.

The Cu_B_^1+^-H_2_O in the E-form completely blocked electron back-leak to Fe_*a*3_^3+^, as described above. This high electron affinity of Cu_B_ minimizes the electron distribution in Fe_*a*3_ in the E-form. Thus, the Cu_B_ in the E-form indirectly increases the electron affinity of Fe_*a*3_. Additionally, the facile proton availability to OA (*i.e.*, OH^−^ at Fe_*a*3_^3+^) from the K-pathway (Fig. S9) *via* the interstitial water could increase the electron affinity of heme *a*_*3*_. These structural findings suggest that the E-form has sufficiently high electron affinity for providing the essentially irreversible E→R transition. Thus, Cu_B_ in the E-form could critically contribute to the high electron affinity of Fe_*a*3_^3+^.

Based on a time-resolved resonance Raman analysis of the A-form (1, 18, 30), it has been proposed that CcO promotes the facile four-electron reduction of the bound O_2_, for minimizing ROS (reactive oxygen species) formation in the cell respiration, by a controlled slow electron donation, from Cu_B_ to the bound O_2_ in the A-form, which lowers the transient appearance of the peroxide-bound form (Fe_*a*3_^3+^-O^−^-O^−^-Cu_B_^2+^) during the CcO reaction. However, the direct structural basis for the slow electron transfer from Cu_B_ to the bound O_2_ has not been obtained yet, although the weak interaction between Cu_B_ and O_2_ bound at Fe_*a*3_ was proposed by the X-ray structural analyses of the A-from models, such as the NO- and CO-bound CcOs (30, 31). The present OxyMb-type structure, showing longer Cu_B_-O_2_ distance than the Fe_*a*3_-O_2_ distance (Fig. 7*B*), supports that the Cu_B_-O_2_ interaction is weak in the A-form. This is a crystallographic confirmation of the above long-standing proposal for the role of Cu_B_ in stabilizing the A-form (1, 31).

### X-ray-structure-based mechanism of the CcO reaction cycle

**Figure 10:**
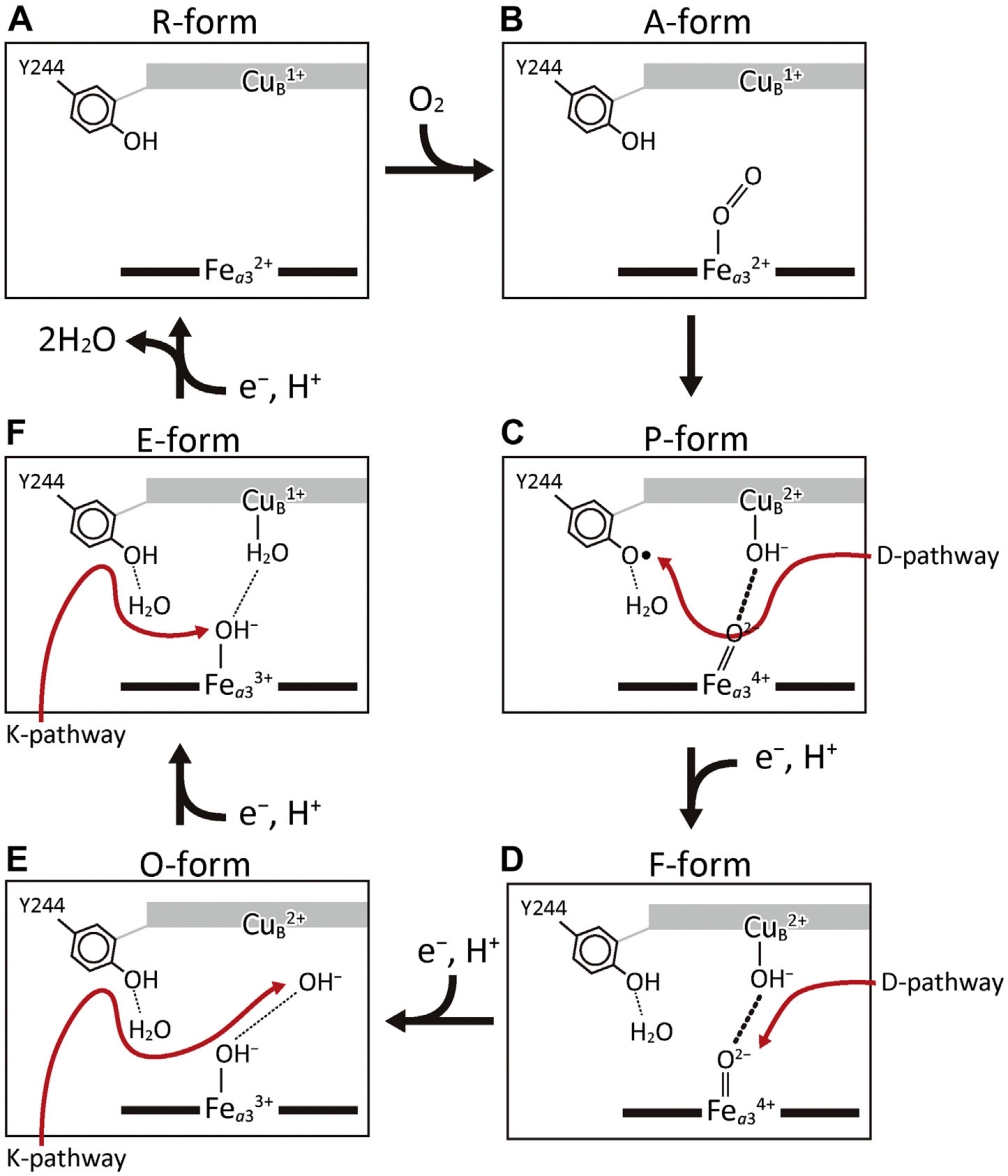
**A schematic representation of the catalytic cycle of bovine CcO based on the X-ray structures of the catalytic intermediate forms of bovine CcO.** The O_2_ reduction site includes Fe_*a*3_, Cu_B_ and Tyr^244^OH group. When the R-form is attained (*A*), the Fe_*a*3_^2+^ receives O_2_ to yield the A-form providing a weak interaction between Cu_B_^1+^ and the bound O_2_, which is displayed by the absence of any line between Cu_B_^1+^ and the O_2_ (*B*). The present OxyMb-type structure supports the weak interaction between O_2_ and Cu_B_^1+^ in the A-form, which has been proposed by resonance Raman and X-ray structural analyses (1, 12, 27). The A-form is relaxed into the P-form in which the bound O_2_ has been completely reduced, giving Fe_*a*3_^4+^ = O^2−^, Cu_B_^2+^-OH^−^, and Tyr^244^ radical with a hydrogen-bonded water (the interstitial water) (*C*). During the P→F transition, the Tyr^244^ radical is transformed to Tyr^244^OH by a proton-coupled electron transfer. The *red arrow* in (*C*) marks the substrate proton transfer pathway from D-pathway to the Tyr^244^ radical through the OH^−^ group at the Cu_B_^2+^, the O^2−^ at the Fe_*a*3_^4+^, and the interstitial water. (The bent structure of HisN-Fe_*a*3_^4+^ = O^2−^ of the P-form (24) is illustrated schematically in *C*.) The second proton-coupled electron transfer to Fe_*a*3_^4+^ = O^2−^ of the F-form from the D-pathway *via* the OH^−^ group, as illustrated by a *red arrow* in (*D*), forms the O-form (*E*). The low-barrier hydrogen bonds between Fe_*a*3_^4+^ = O^2−^ and Cu_B_^2+^-OH^−^ in both the P- and F-forms are shown by *thick dotted lines* in *C* and *D*. The unusually long distance between Cu_B_^2+^ and OH^−^ is illustrated by deleting any lines between them (*E*). In the O to E transition (*E* to *F*), the substrate protons are transferred from K-pathway to the OH^−^ group near the Cu_B_^2+^ through the interstitial water and the OH^−^ group at the Fe_*a*3_^3+^ (*E*), giving Cu_B_^1+^-H_2_O (*F*) as illustrated by *red arrows* (*E*). In the O- and E-forms, normal hydrogen bonds are detectable between the two oxygen atoms as illustrated by *thin dotted lines* (*E* and *F*). The forth proton-coupled electron transfer through K-pathway and the interstitial water illustrated by a *red arrow* (*F*) regenerates the R-form (*A*), releasing two water molecules. The interstitial water molecules, hydrogen-bonded to Tyr^244^, in *C*–*F* could be transferred reversibly from a storage site near the O_2_-reduction site, not from the bulk water phase.

The present X-ray structural findings for the catalytic intermediate forms, O, E, and R, together with previously reported P- and F-forms, provide the structural basis for a possible catalytic cycle of CcO as outlined in Figure 10. The R-form including Cu_B_^1+^ and Fe_*a*3_^2+^ and Tyr^244^-OH group (Fig. 10*A*) receives an O_2_ molecule to form the A-form (Fig. 10*B*). The OxyMb type structure is tentatively given as that of the A-form in this scheme. The A-form is spontaneously transformed to the P-form in which the bound O_2_ has been completely reduced, yielding Fe_*a*3_^4+^ = O^2−^, Cu_B_^2+^-OH^−^, and Tyr^244^-O• with the interstitial water (Fig. 10*C*). The weak interaction between Cu_B_^1+^ and the distal oxygen atoms of the bound O_2_ in the A-form (Fig. 10*B*) provides the stability of the A-form, which induces the A→P transition without any peroxide intermediate (1, 30, 31). It has been widely accepted that, in the P→F transition, the Tyr^244^-O• is transformed to Tyr^244^OH by a proton-coupled electron transfer in which electrons and protons are from heme *a* and D-pathway, respectively (1, 10, 11). The high-resolution X-ray structures of the P- and F-forms (23) suggest that OH^−^ group of the Cu_B_^2+^-OH^−^, Fe^4+^ = O^2−^ group of the Fe_*a*3_^4+^ = O^2−^ and the interstitial water facilitate the proton-transfer pathway to the Tyr-O•, as schematically illustrated with a red arrow in Figure 10*C*. By another proton transfer to Fe_*a*3_^4+^ = O^2−^ of the F-form through the D-pathway *via* OH^−^ group of the Cu_B_^2+^-OH^−^, coupled with electron transfer to Fe_*a*3_^4+^, the O-form is generated (Fig. 10, *D* and *E*). In the O to E transition, the substrate (*i.e.*, water-forming) protons are transferred from K-pathway to the OH^−^ near the Cu_B_^2+^ site *via* the interstitial water and OH^−^ group of Fe_*a*3_^3+^-OH^−^ (Fig. 10*E*), giving Cu_B_^1+^-H_2_O (Fig. 10*F*). This proton transfer is coupled with an electron transfer to Cu_B_^2+^. An additional electron-coupled proton transfer through K-pathway and the interstitial water (Fig. 10*F*) regenerates the R-form (Fig. 10*A*), releasing two water molecules. The strong electron affinity of the Cu_B_ site, detected by the present X-ray structural analysis, is implemental to the essentially irreversible O→E and E→R transitions.

### The open/closed transition of the water channel of the H-pathway during the catalytic turnover

Based on the X-ray structures of various static CcO forms (not involved in the catalytic cycle) (9, 30, 31, 32), it has been proposed that the unidirectional proton transport across the CcO molecule is facilitated by the closure of the water channel. To verify this proposal, high-resolution X-ray structures of all catalytic intermediate forms, involved in the proton pumping process, P, F, O, and E, were determined and reported in the present and previous studies (23). These results indicate that active unidirectional proton transport through the H-pathway is facilitated by water channel closure. An XFEL study on photolysis of a CcO-CO complex suggests that the channel closure upon O_2_ binding is necessary for the complete prevention of back-leak of pumping protons from the water channel (30). The channel closure observed for the Oxy-Mb type form here supports the above proposal based on a model study using the respiratory inhibitor, CO.

The present re-refined fully reduced form structure revealed that the reduced- and oxidized-type structures of the residue 380 to 385 region (or the open and closed structures) are in an equilibrium state in the R-form, indicating low energy costs for open/close transition of the water channel. However, the 48 to 55 residue region is completely fixed in the reduced-type structure. Asp^51^ in this region facilitates the redox-coupled proton migration at the P-side end of the H-pathway, when the water channel of the H-pathway is closed. This residue in the fully reduced form is accessible only to the P-side. Since the water channel is in the open state in the fully reduced form, fixing the 48 to 55 region in the reduced-type structure in the fully reduced form completely would be critical for preventing the proton back-leak from the P-side to the N-side.

## Experimental procedures

### Preparation of the fully reduced CcO crystals exposed by excess O_2_

Resting-oxidized bovine heart CcO crystals were prepared as previously described (16). Isomorphous crystals were efficiently prepared as follows: The final medium composition for the crystals (40 mM sodium phosphate buffer pH 5.7, 0.2% decylmaltoside, 8% polyethylene glycol 4000, and 40% ethylene glycol) was attained by 50 stepwise manual exchanges from the initial medium composed of 40 mM sodium phosphate buffer pH 6.5, 0.2% decylmaltoside, 1% polyethylene glycol 4000, and 2% ethylene glycol in which the crystals are stable at 4 °C. The crystals were reduced by soaking the crystals in the final medium containing 5 mM dithionite and an O_2_-scavenging system composed of catalase (0.5 μM), glucose (5 mM), and glucose oxidase (1 μM). When absorption spectral increase in the α-band region was completed, the soaking medium was replaced with an O_2_-saturated medium for initiation of the oxidation of the reduced CcO in the final medium. Before freezing at 90K, the absorption spectral changes were followed after O_2_ treatment as previously described (23).

### The structure determination procedure of a model with a singular structure

Initial phase angles of structure factors up to a resolution of 5.0 Å for three data sets of IO10, IOF20, and IO80 were calculated by the molecular replacement (MR) method (33) using a fully oxidized structure previously determined at 1.5 Å resolution (PDB ID code 5B1A) (33) after removing nonprotein molecules, including peroxide ligands, water molecules, lipids, and detergents. The phases were extended to the highest resolutions of each data set by density modification (34) coupled with non-crystallographic symmetry (NCS) averaging (35, 36) using the CCP4 program DM (37). These phase extension procedures were the same as those applied to the previous crystal structure analysis of the CcO intermediate form (23). The resultant phase angles (α_MR/DM_) were used to calculate the electron density map (MR/DM map) with Fourier coefficients |*F*_o_| *exp*(iα_MR/DM_), where |*F*_o_| is the amplitude of the observed structure factor.

A structural model of reduced CcO previously determined at 1.6 Å resolution (9) was superposed well on the MR/DM map of IO10. Those of IO20 and IO80 were successfully traced by a structural model of oxidized CcO (PDB code 5B1A) determined at 1.5 Å resolution. Structure refinements were performed using alternating rounds of model building with program COOT (38) and restrained refinement with phenix.refine (39). The phenix refinement was performed without simulated annealing procedure unless otherwise stated. An asymmetric unit of the unit cell contains two monomers of A and B, each consisting of 13 different protein subunits. Each monomer in the asymmetric unit, related by NCS, was assigned to a single group for TLS refinement. The anisotropic temperature factors for the zinc, copper, iron, and magnesium atoms were imposed on the calculated structure factors. Molecules of water, ethylene glycol, lipids, and detergents were located on the MR/DM map and *F*_o_−*F*_c_ maps composed of the phases calculated using atomic parameters of protein atoms and other molecules that had been determined. Refinement statistics are listed in Table 4.

**Table 4:** Structure refinement statistics

Crystals (PDB ID codes)	IO10 (7D5X)	IO20 (7D5W)	IO80 (7CP5)	Fully reduced form^a^ (5B1B)
Resolution (Å)	39.70–1.74 (1.76–1.74)	39.92–1.84 (1.86–1.84)	39.91–1.76 (1.78–1.76)	39.90–1.60 (1.62–1.60)
Number of reflections in work set	629,954 (20,340)	533,026 (17,436)	609,364 (20,207)	794,473 (26,295)
Number of reflections in test set	33,287 (1074)	28,279 (893)	32,218 (1043)	41,978 (1408)
*R*_work_ (%)	15.59 (23.56)	15.31 (21.47)	16.03 (25.78)	16.84 (23.68)
*R*_free_ (%)	18.36 (26.28)	18.41 (24.74)	18.86 (28.79)	19.10 (27.18)
Non-hydrogen atom numbers				
Total	33,923	34,545	34,258	34,425
Proteins	29,171	29,284	29,381	29,067
Lipids	1297	1287	1133	1264
Detergents	509	639	482	531
Water	2612	2975	2968	3305
Ethylene glycol	324	320	284	248
Phosphate	10	10	10	10
Ligands	4	8	8	0
R.m.s. deviations				
Bonds (Å)	0.020	0.018	0.017	0.019
Angles (°)	1.85	1.64	1.59	1.63
Ramachandran statistics				
Favoured (%)	96.71	96.88	96.99	96.97
Allowed (%)	2.98	2.63	2.58	2.80
Outliers (%)	0.32	0.49	0.43	0.23
Clashscore	6.69	6.65	5.43	4.65
Average *B*-factor (Å^2^)				
Overall	40.5	41.1	40.3	38.5
Protein (A^b^)	35.7	35.1	34.7	32.2
Protein (B^b^)	40.4	40.4	40.7	38.1
Others	55.7	59.3	55.5	56.5

a The atomic parameters were revised by the present work.

b A and B indicate monomers A and B, respectively.

## Data availability

The atomic parameters and structure factors (PDB ID codes 7D5X, 7D5W, and 7CP5, for IO10, IO20, and IO80, respectively) have been deposited in the Protein Data Bank (http://wwpdb.org/). The atomic parameters of the fully reduced form (PDB ID code 5B1B) in the protein data bank were revised. All the other data are contained within this manuscript.

## Supporting information

This article contains supporting information (23, 25, 26, 27).

## Conflict of interest

Each of the authors of this manuscript declares that they have no conflicts of interest with regard to the contents of this article. The authors state that none of the new findings in this manuscript have been published or are under consideration for publication elsewhere.

## References

[1] Yoshikawa S., Shimada A. (2015). Reaction mechanism of cytochrome *c* oxidase. Chem. Rev..

[2] Wikström M., Krab K., Sharma V. (2018). Oxygen activation and energy conservation by cytochrome *c* oxidase. Chem. Rev..

[3] Bloch D., Belevich I., Jasaitis A., Ribacka C., Puustinen A., Verkhovsky M.I., Wikström M. (2004). The catalytic cycle of cytochrome *c* oxidase is not the sum of its two halves. Proc. Natl. Acad. Sci. U. S. A..

[4] Tsukihara T., Shimokata K., Katayama Y., Shimada H., Muramoto K., Aoyama H., Mochizuki M., Shinzawa-Itoh K., Yamashita E., Yao M., Ishimura Y., Yoshikawa S. (2003). The low-spin heme of cytochrome *c* oxidase as the driving element of the proton-pumping process. Proc. Natl. Acad. Sci. U. S. A..

[5] Yoshikawa S., Shinzawa-Itoh K., Nakashima R., Yaono R., Yamashita E., Inoue N., Yao M., Fei M.J., Libeu C.P., Mizushima T., Yamaguchi H., Tomizaki T., Tsukihara T. (1998). Redox-coupled crystal structural changes in bovine heart cytochrome *c* oxidase. Science.

[6] Shimokata K., Katayama Y., Murayama H., Suematsu M., Tsukihara T., Muramoto K., Aoyama H., Yoshikawa S., Shimada H. (2007). The proton pumping pathway of bovine heart cytochrome *c* oxidase. Proc. Natl. Acad. Sci. U. S. A..

[7] Lee H.M., Das T.K., Rousseau D.L., Mills D., Ferguson-Miller S., Gennis R.B. (2000). Mutations in the putative H-channel in the cytochrome *c* oxidase from *Rhodobacter sphaeroides* show that this channel is not important for proton conduction but reveal modulation of the properties of heme a. Biochemistry.

[8] Lepp H., Salomonsson L., Zhu J.-P., Gennis R.B., Brzezinski P. (2008). Impaired proton pumping in cytochrome *c* oxidase upon structural alteration of the D pathway. Biochim. Biophys. Acta.

[9] Yano N., Muramoto K., Shimada A., Takemura S., Baba J., Fujisawa H., Mochizuki M., Shinzawa-Itoh K., Yamashita E., Tsukihara T., Yoshikawa S. (2016). The Mg^2+^-containing water cluster of mammalian cytochrome *c* oxidase collects four pumping proton equivalents in each catalytic cycle. J. Biol. Chem..

[10] Proshlyakov D.A., Pressler M.A., DeMaso C., Leykam J.F., DeWitt D.L., Babcock G.T. (2000). Oxygen activation and reduction in respiration: Involvement of redox-active tyrosine 244. Science.

[11] Yu M.A., Egawa T., Shinzawa-Itoh K., Yoshikawa S., Guallar V., Yeh S.-R., Rousseau D.L., Gerfen G.J. (2012). Two tyrosyl radicals stabilize high oxidation states in cytochrome *c* oxidase for efficient energy conservation and proton translocation. J. Am. Chem. Soc..

[12] Wiertz F.G.M., Richter O.-M.H., Ludwig B., de Vries S. (2007). Kinetic resolution of a tryptophan-radical intermediate in the reaction cycle of paracoccus denitrificans cytochrome *c* oxidase. J. Biol. Chem..

[13] Belevich I., Bloch D.A., Belevich N., Wikström M., Verkhovsky M.I. (2007). Exploring the proton pump mechanism of cytochrome *c* oxidase in real time. Proc. Natl. Acad. Sci. U. S. A..

[14] Moody A.J. (1996). “As prepared” forms of fully oxidised haem/Cu terminal oxidases. Biochim. Biophys. Acta.

[15] Aoyama H., Muramoto K., Shinzawa-Itoh K., Hirata K., Yamashita E., Tsukihara T., Ogura T., Yoshikawa S. (2009). A peroxide bridge between Fe and Cu ions in the O_2_ reduction site of fully oxidized cytochrome *c* oxidase could suppress the proton pump. Proc. Natl. Acad. Sci. U. S. A..

[16] Mochizuki M., Aoyama H., Shinzawa-Itoh K., Usui T., Tsukihara T., Yoshikawa S. (1999). Quantitative reevaluation of the redox active sites of crystalline bovine heart cytochrome *c* oxidase. J. Biol. Chem..

[17] Sakaguchi M., Shinzawa-Itoh K., Yoshikawa S., Ogura T. (2010). A resonance Raman band assignable to the O-O stretching mode in the resting oxidized state of bovine heart cytochrome *c* oxidase. J. Bioenerg. Biomembr..

[18] Ogura T., Takahashi S., Hirota S., Shinzawa-Itoh K., Yoshikawa S., Appelman E.H., Kitagawa T. (1993). Time-resolved resonance Raman elucidation of the pathway for dioxygen reduction by cytochrome *c* oxidase. J. Am. Chem. Soc..

[19] Ishigami I., Lewis-Ballester A., Echelmeier A., Brehm G., Zatsepin N.A., Grant T.D., Coe J.D., Lisova S., Nelson G., Zhang S., Dobson Z.F., Boutet S., Sierra R.G., Batyuk A., Fromme P. (2019). Snapshot of an oxygen intermediate in the catalytic reaction of cytochrome *c* oxidase. Proc. Natl. Acad. Sci. U. S. A..

[20] Nango E., Royant A., Kubo M., Nakane T., Wickstrand C., Kimura T., Tanaka T., Tono K., Song C., Tanaka R., Arima T., Yamashita A., Kobayashi J., Hosaka T., Mizohata E. (2016). A three-dimensional movie of structural changes in bacteriorhodopsin. Science.

[21] Tosha T., Nomura T., Nishida T., Saeki N., Okubayashi K., Yamagiwa R., Sugahara M., Nakane T., Yamashita K., Hirata K., Ueno G., Kimura T., Hisano T., Muramoto K., Sawai H. (2017). Capturing an initial intermediate during the P450nor enzymatic reaction using time-resolved XFEL crystallography and caged-substrate. Nat. Commun..

[22] Hirata K., Shinzawa-Itoh K., Yano N., Takemura S., Kato K., Hatanaka M., Muramoto K., Kawahara T., Tsukihara T., Yamashita E., Tono K., Ueno G., Hikima T., Murakami H., Inubushi Y. (2014). Determination of damage-free crystal structure of an X-ray-sensitive protein using an XFEL. Nat. Methods.

[23] Shimada A., Etoh Y., Kitoh-Fujisawa R., Sasaki A., Shinzawa-Itoh K., Hiromoto T., Yamashita E., Muramoto K., Tsukihara T., Yoshikawa S. (2020). X-ray structures of catalytic intermediates of cytochrome *c* oxidase provide insights into its O_2_ activation and unidirectional proton-pump mechanisms. J. Biol. Chem..

[24] Vojtechovský J., Chu K., Berendzen J., Sweet R.M., Schlichting I. (1999). Crystal structures of myoglobin-ligand complexes at near-atomic resolution. Biophys. J..

[25] Olmstead M.M., Musker W.K., Kessler R.M. (1982). Differences in the coordinating ability of water, perchlorate and tetrafluoroborate toward copper(I). The x-ray crystal structures of [Cu(1,4-thioxane)_3_OClO_3_], [Cu(1,4-thioxane)_3_OH_2_]BF_4_ and [Cu(1,4-thioxane)_4_BF_4_. Transit. Met. Chem..

[26] Štěpnička P., Císařová I. (2013). Selective borane reduction of phosphinoferrocene carbaldehydes to phosphinoalcohol–borane adducts. The coordination behaviour of 1-(diphenylphosphino)-1′-(methoxymethyl)ferrocene, a new ferrocene O,P-hybrid donor prepared from such an adduct. Dalt. Trans..

[27] Dai Y., Zhang Y., Tian J., Liu Z. (2009). Aqua-bis(triphenyl-phosphine-κP)copper(I) tetra-fluoridoborate. Acta Crystallogr. Sect. E. Struct. Rep. Online.

[28] Wikström M. (2012). Active site intermediates in the reduction of O_2_ by cytochrome oxidase, and their derivatives. Biochim. Biophys. Acta.

[29] Einarsdóttir O., Szundi I., Van Eps N., Sucheta A. (2002). P(M) and P(R) forms of cytochrome *c* oxidase have different spectral properties. J. Inorg. Biochem..

[30] Shimada A., Kubo M., Baba S., Yamashita K., Hirata K., Ueno G., Nomura T., Kimura T., Shinzawa-Itoh K., Baba J., Hatano K., Eto Y., Miyamoto A., Murakami H., Kumasaka T. (2017). A nanosecond time-resolved XFEL analysis of structural changes associated with CO release from cytochrome *c* oxidase. Sci. Adv..

[31] Muramoto K., Ohta K., Shinzawa-Itoh K., Kanda K., Taniguchi M., Nabekura H., Yamashita E., Tsukihara T., Yoshikawa S. (2010). Bovine cytochrome *c* oxidase structures enable O_2_ reduction with minimization of reactive oxygens and provide a proton-pumping gate. Proc. Natl. Acad. Sci. U. S. A..

[32] Shimada A., Hatano K., Tadehara H., Yano N., Shinzawa-Itoh K., Yamashita E., Muramoto K., Tsukihara T., Yoshikawa S. (2018). X-ray structural analyses of azide-bound cytochrome *c* oxidases reveal that the H-pathway is critically important for the proton-pumping activity. J. Biol. Chem..

[33] Rossmann M.G., Blow D.M. (1962). The detection of sub-units within the crystallographic asymmetric unit. Acta Crystallogr..

[34] Wang B.C. (1985). Resolution of phase ambiguity in macromolecular crystallography. Methods Enzymol..

[35] Bricogne G. (1974). Geometric sources of redundancy in intensity data and their use for phase determination. Acta Crystallogr. A.

[36] Bricogne G. (1976). Methods and programs for direct-space exploitation of geometric redundancies. Acta Crystallogr. A.

[37] Cowtan K. (1994). DM: An automated procedure for phase improvement by density modification. Jt. CCP4 ESF-EACBM Newsl. Protein Crystallogr..

[38] Emsley P., Lohkamp B., Scott W.G., Cowtan K. (2010). Features and development of Coot. Acta Crystallogr. D Biol. Crystallogr..

[39] Afonine P.V., Grosse-Kunstleve R.W., Echols N., Headd J.J., Moriarty N.W., Mustyakimov M., Terwilliger T.C., Urzhumtsev A., Zwart P.H., Adams P.D. (2012). Towards automated crystallographic structure refinement with *phenix.refine*. Acta Crystallogr. D Biol. Crystallogr..

