# Supplementary material for: Critical roles of the CuB site in efficient proton pumping as revealed by crystal structures of mammalian cytochrome c oxidase catalytic intermediates
Source: J Biol Chem. 2021 Jul 15;297(3):100967. doi: 10.1016/j.jbc.2021.100967 (PMC8390519; doi:10.1016/j.jbc.2021.100967)
Supplement: Supplemental Figures S1–S9, Table S1 and Supporting Text 1–4 [file mmc2.pdf]

Supporting Information

Critical roles of the Cu_B site in efficient proton pumping as revealed by crystal structures of mammalian cytochrome *c* oxidase catalytic intermediates

Atsuhiro Shimada, Fumiyoshi Hara, Kyoko Shinzawa-Itoh, Nobuko Kanehisa, Eiki Yamashita, Kazumasa Muramoto, Tomitake Tsukihara, and Shinya Yoshikawa

The material includes:

1. Figures S1–S9
2. Table S1
3. Supporting Text 1-4
4. Movie S1

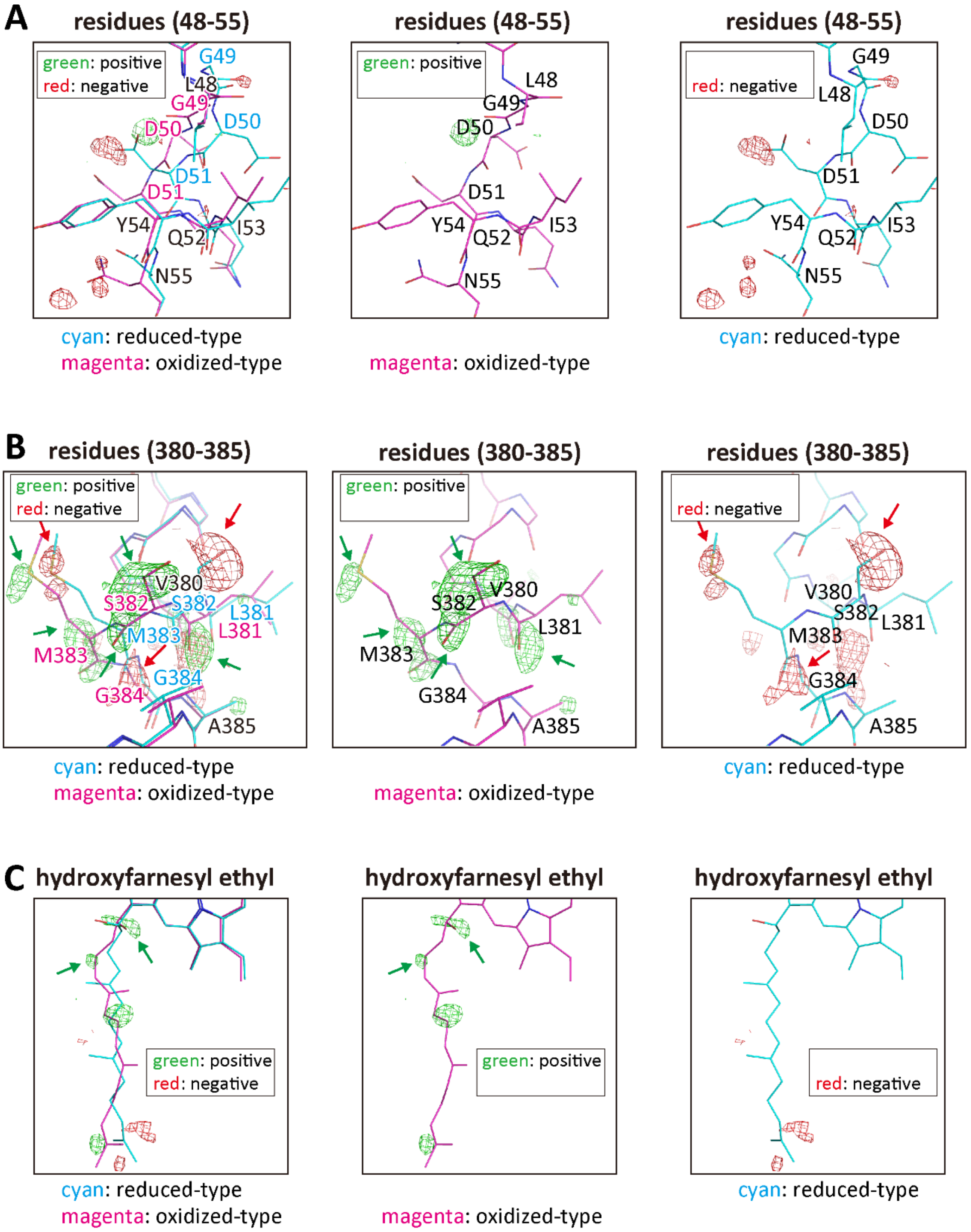

Figure. S1. Minor component searches for the refined fully-reduced form by calculating the F_o-F_c maps. *A*, the F_o-F_c map, drawn at 3.0 σ, for the region including residues 48-55. The left panel: both negative (red) and positive (green) densities including the atomic models of the oxidized (magenta)- and reduced (cyan)-type structures. The panel is given also in Fig. 5A in

the text. The center panel: the positive densities (green) in the left panel are compared with the atomic model of the oxidized (magenta)-type structure. The right panel: the negative densities (red) in the left panel are compared with the atomic model of the reduced (cyan)-type structure. *B*, the F_o-F_c maps, drawn at 3.0 σ, for the region including residues 380-385. The left panel: both negative (red) and positive (green) densities including the atomic models of the oxidized (magenta)- and reduced (cyan)-type structures. Representative positive density cages on the oxidized-type model are indicated by green arrows, and representative negative density cages on the reduced-type model are indicated by red arrows. The panel is given also in Fig. 5B in the text. The center panel: the positive densities (green) in the left panel are compared with the atomic model of the oxidized (magenta)-type structure. Representative positive density cages on the oxidized-type model are indicated by green arrows. The right panel: the negative densities (red) in the left panel are compared with the atomic model of the reduced (cyan)-type structure. Representative negative density cages on the reduced-type model are indicated by red arrows. *C*, the F_o-F_c maps, drawn at 3.0 σ, for the region including the hydroxyfarnesyl ethyl group of heme *a*. The left panel: both negative (red) and positive (green) densities including the atomic models of the oxidized (magenta)- and reduced (cyan)-type structures. Representative positive density cages on the oxidized-type model are indicated by green arrows. The panel is given also in Fig. 5C in the text. The center panel: the positive density cages (green) in the left panel with the atomic model of the oxidized (magenta)-type structure. The right panel: the negative density cages (red) in the left panel with the atomic model of the reduced (cyan)-type structure.

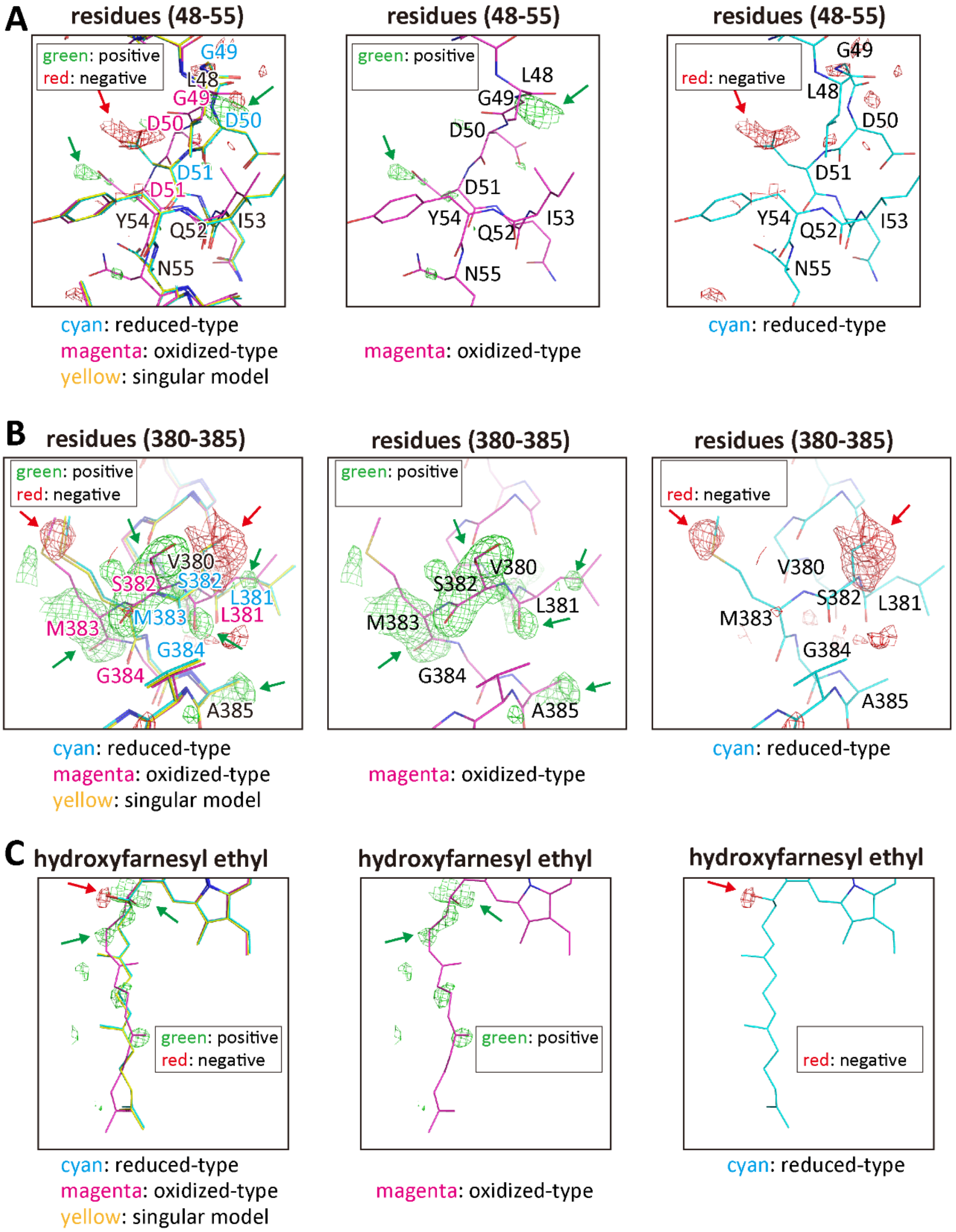

Figure. S2. Minor component searches for the electron density obtained from the data set IO10 by calculating the F_o-F_c maps. Representative negative and positive density cages on the reduced-type and oxidized-type models in each panel are indicated by green and red arrows respectively. *A*, the F_o-F_c map, drawn at 3.0 σ, for the region including residues 48-55. The left panel: both negative (red) and positive (green) density cages including the atomic models of the

oxidized (magenta)- and reduced (cyan)-type structures and those of the singular structures (yellow) which are closely similar to those of the reduced-type structure as described in the text. The center panel: the positive density cages (green) in the left panel with the atomic model of the oxidized (magenta)-type structure. The right panel: the negative density cages (red) in the left panel with the atomic model of the reduced (cyan)-type structure. *B*, the F_o-F_c maps, drawn at 3.0 σ, for the region including residues 380-385. The left panel: both negative (red) and positive (positive) densities including the atomic models of the oxidized (magenta)- and reduced (cyan)-type structures and those of the singular structures (yellow). The center panel: the positive densities (green) given in the left panel with the atomic model of the oxidized (magenta)-type structure. The right panel: the negative densities (red) given in the left panel with the atomic model of the reduced (cyan)-type structure. *C*, the F_o-F_c maps, drawn at 3.0 σ, for the region including the hydroxyfarnesyl ethyl group of heme *a*. The left panel: both negative (red) and positive (green) densities including the atomic models of the oxidized (magenta)- and reduced (cyan)-type structures and those of the singular structures (yellow). The center panel: the positive density cage (green) in the left panel with the atomic model of the oxidized (magenta)-type structure. The right panel: the negative peaks (red) given in the left panel with the atomic model of the reduced (cyan)-type structure.

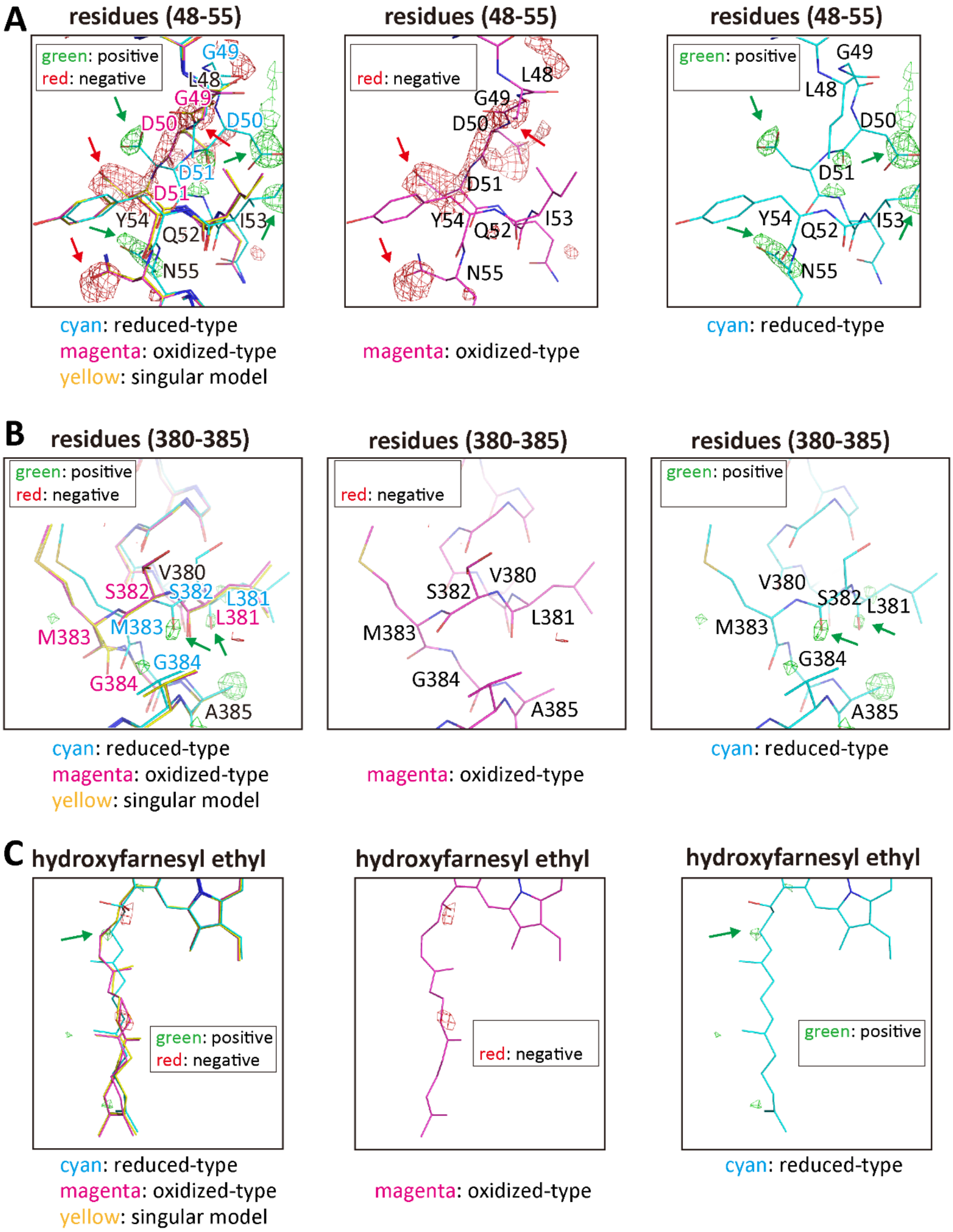

Figure. S3. Minor component searches for the electron density obtained from the data set IO20 by calculating the F_o-F_c maps. Representative negative and positive density cages on the oxidized- and reduced-type models in each panel are indicated by red and green arrows respectively. *A*, the F_o-F_c map, drawn at 3.0 σ, for the region including residues 48-55. The left

panel: both negative (red) and positive (green) density cages including the atomic models of the oxidized (magenta)- and reduced (cyan)-type structures and those of the singular structures (yellow) which are closely similar to those of the oxidized-type structure as described in the text. The center panel: the negative density cages (red) given in the left panel with the atomic model of the oxidized (magenta)-type structure. The right panel: the positive density cages (green) given in the left panel with the atomic model of the reduced (cyan)-type structure. *B*, the F_o-F_c maps, drawn at 3.0 σ, for the region including residues 380-385. The left panel: positive (green) densities including the atomic models of the oxidized (magenta)- and reduced (cyan)-type structures and those of the singular structures (yellow). No significant negative density is detectable at 3.0 σ. The center panel: the atomic model of the oxidized (magenta)-type structure. The right panel: the positive density cages (green) given in the left panel with the atomic model of the reduced (cyan)-type structure. *C*, the F_o-F_c maps, drawn at 3.0 σ, for the region including the hydroxyfarnesyl ethyl group of heme *a*. The left panel: both negative (red) and positive (green) density cages including the atomic models of the oxidized (magenta)- and reduced (cyan)-type structures and those of the singular structures (yellow). The center panel: the negative density cages (red) given in the left panel with the atomic model of the oxidized (magenta)-type structure. The right panel: the positive density cages (green) given in the left panel with the atomic model of the reduced (cyan)-type structure.

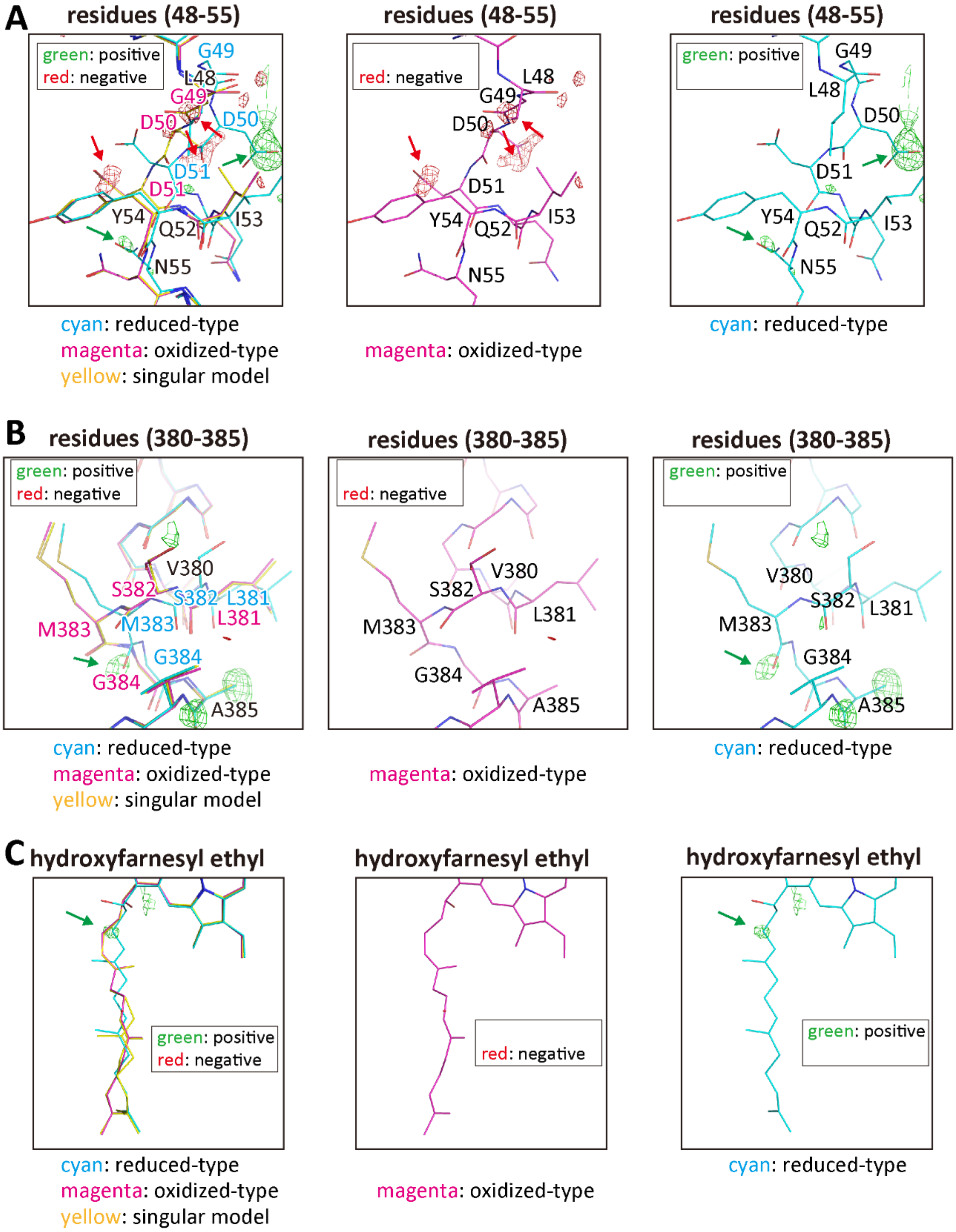

Figure. S4. Minor component searches for the electron density obtained from the data set IO80 by calculating the F_o-F_c maps. Representative negative and positive density cages on the oxidized- and reduced-type models in each panel are indicated by red and green arrows respectively. *A*, the F_o-F_c map, drawn at 3.0 σ, for the region including residues 48-55. The left

panel: both negative (red) and positive (green) density cages including the atomic models of the oxidized (magenta)- and reduced (cyan)-type structures and those of the singular structures (yellow). The center panel: the negative density cages (red) given in the left panel with the atomic model of the oxidized (magenta)-type structure. The right panel: the positive density cages (green) given in the left panel with the atomic model of the reduced (cyan)-type structure. *B*, the F_o-F_c maps, drawn at 3.0 σ, for the region including residues 380-385. The left panel: positive (green) densities including the atomic models of the oxidized (magenta)- and reduced (cyan)-type structures and those of the singular structures (yellow). No significant negative density is detectable at 3.0 σ. The center panel: the atomic model of the oxidized (magenta)-type structure. The right panel: the positive density cages (green) given in the left panel with the atomic model of the reduced (cyan)-type structure. *C*, the F_o-F_c maps, drawn at 3.0 σ, for the region including the hydroxyfarnesyl ethyl group of heme *a*. The left panel: positive (green) density cages including the atomic models of the oxidized (magenta)- and reduced (cyan)-type structures and those of the singular structures (yellow). No significant negative density is detectable at 3.0 σ. The center panel: the atomic model of the oxidized (magenta)-type structure. The right panel: the positive density cages (green) given in the left panel with the atomic model of the reduced (cyan)-type structure.

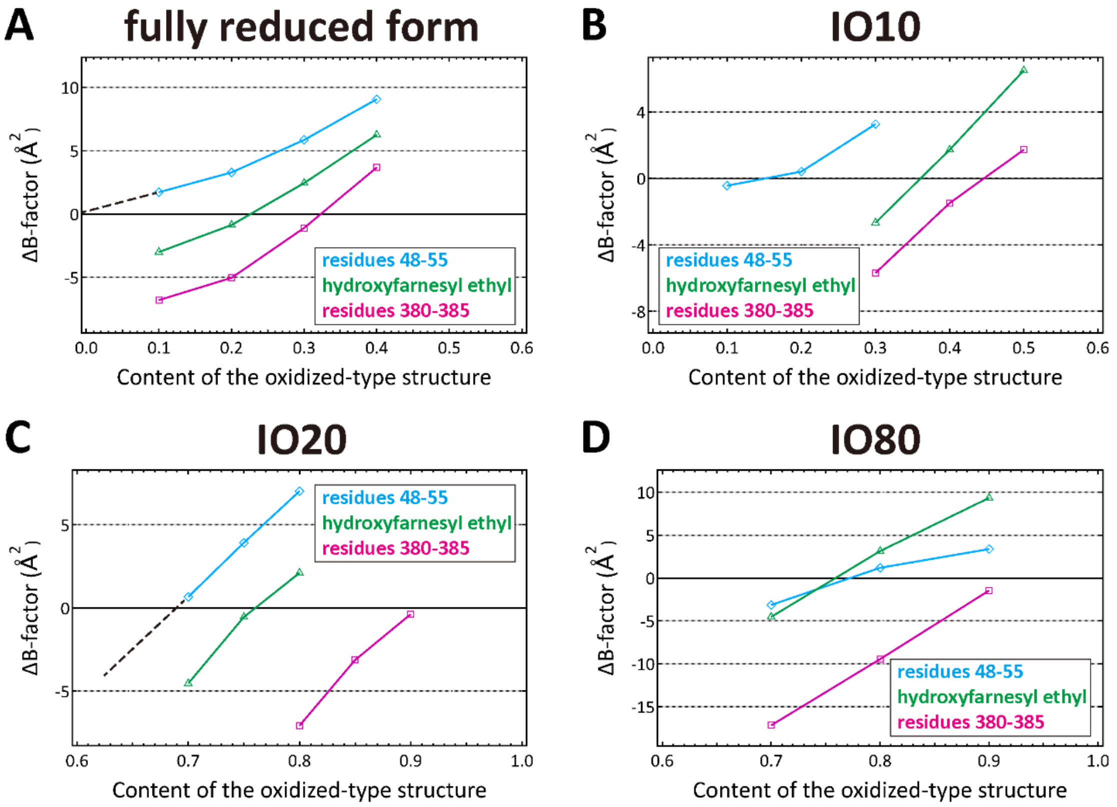

Figure S5. Effect of content of the oxidized-type structure on the average *B*-factor values of the refined structures of both the oxidized- and reduced-type structures for the monomer B. The effect is shown by the difference between the average *B*-factor values of the refined oxidized- and reduced-type structures (ΔB), defined as follows:

ΔB = average *B*-factor value of the refined reduced-type structure - average *B*-factor value of the refined oxidized-type structure.

The ΔB values, determined at various contents of the reduced-type structure, are plotted against the content of the oxidized-type structure included for the hydroxyfarnesyl ethyl group of heme *a* in green, and for residues 48–55 and 380–385, in blue and red plots, respectively. *A, B, C,* and *D,* the ΔB plots for the fully reduced form, IO10, IO20, and IO80, respectively.

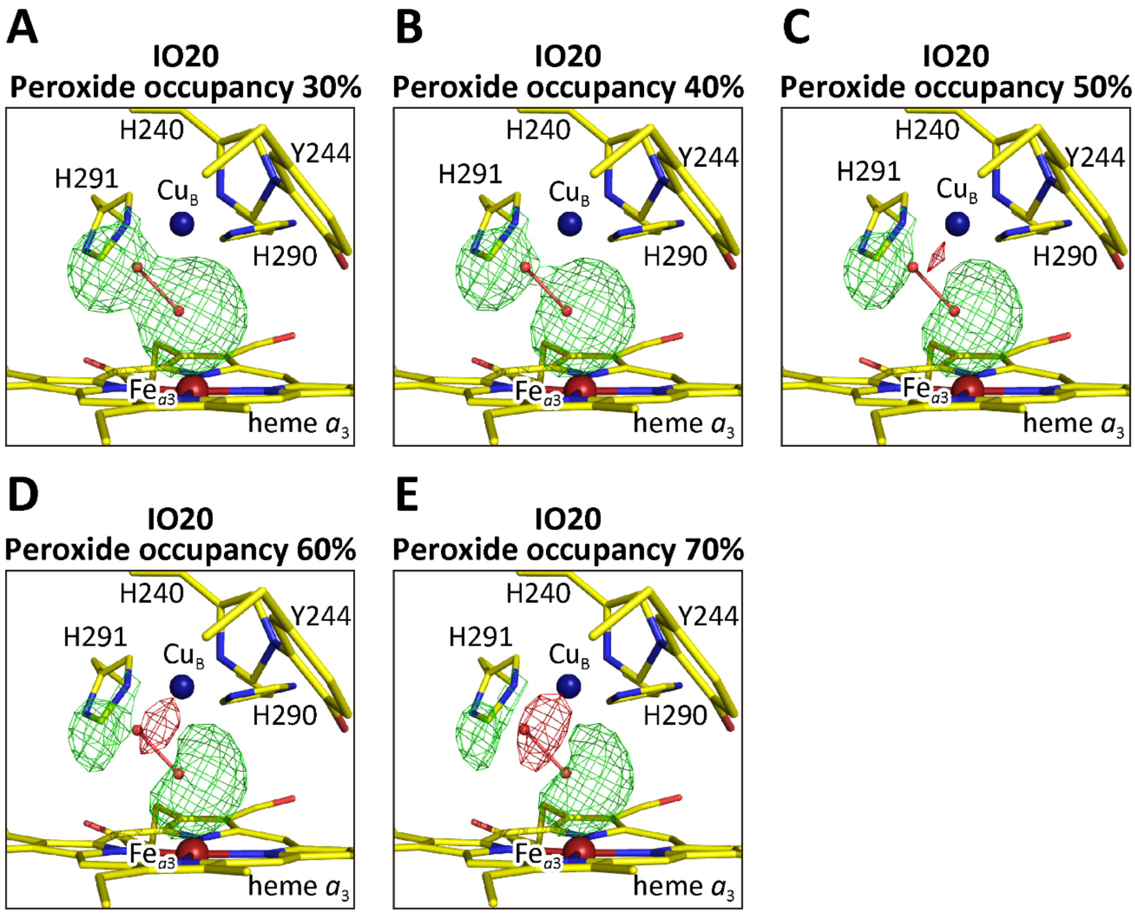

Figure S6. ***Examination of occupancy of the peroxide ligand in IO20 of monomer A.*** The F_o-F_c maps for the electron densities in the O_2-reduction site calculated assuming the occupancies of the peroxide ligand of 30 % (*A*), 40 % (*B*), 50 % (*C*), 60 % (*D*), and 70 % (*E*). The red sticks denote the location of the peroxide ligand. Positive and negative densities are drawn at 2.54 σ by green and red cages, respectively.

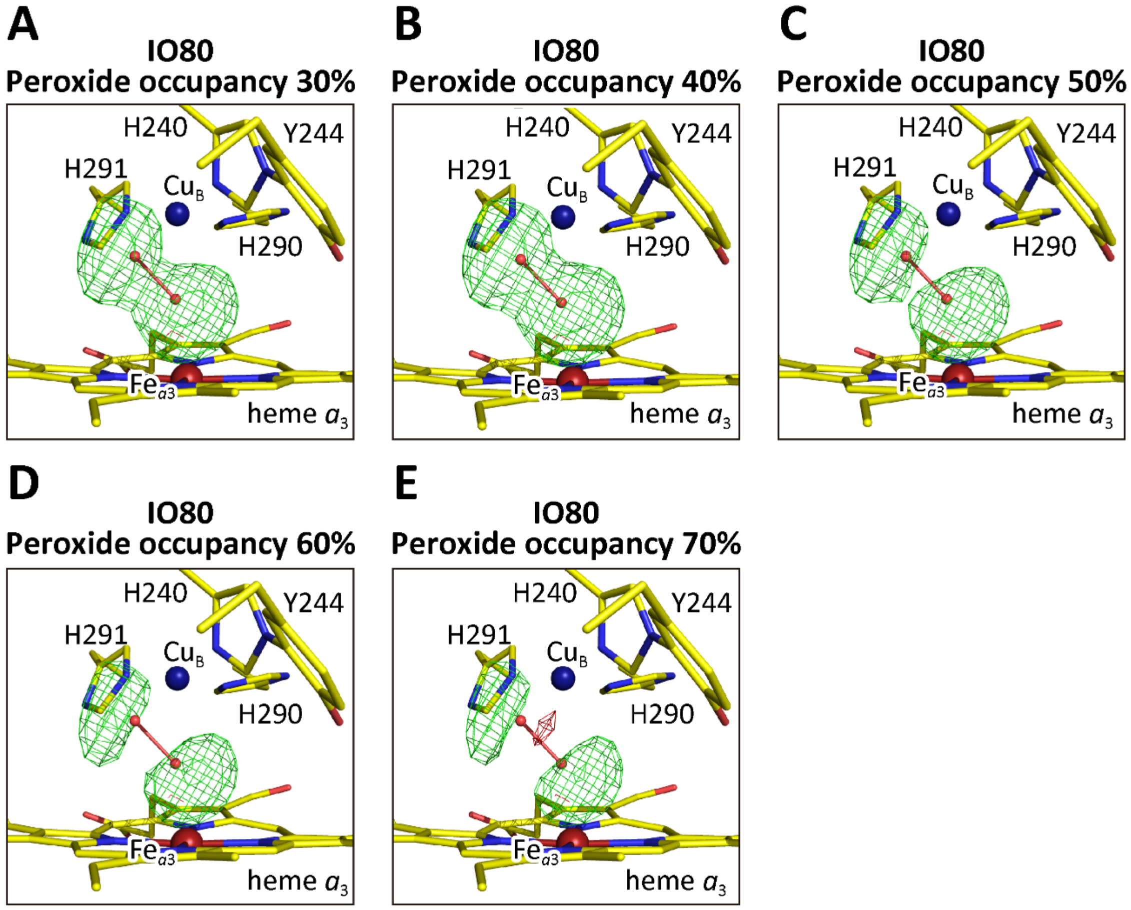

Figure S7. ***Examination of occupancy of the peroxide ligand in IO80 of monomer A.*** The F_o-F_c maps for the electron densities in the O_2-reduction site calculated assuming the occupancies of the peroxide ligand of 30 % (*A*), 40 % (*B*), 50 % (*C*), 60 % (*D*) and 70 % (*E*). The red sticks denote the location of the peroxide ligand. Positive and negative densities are drawn at 2.55 σ by green and red cages, respectively.

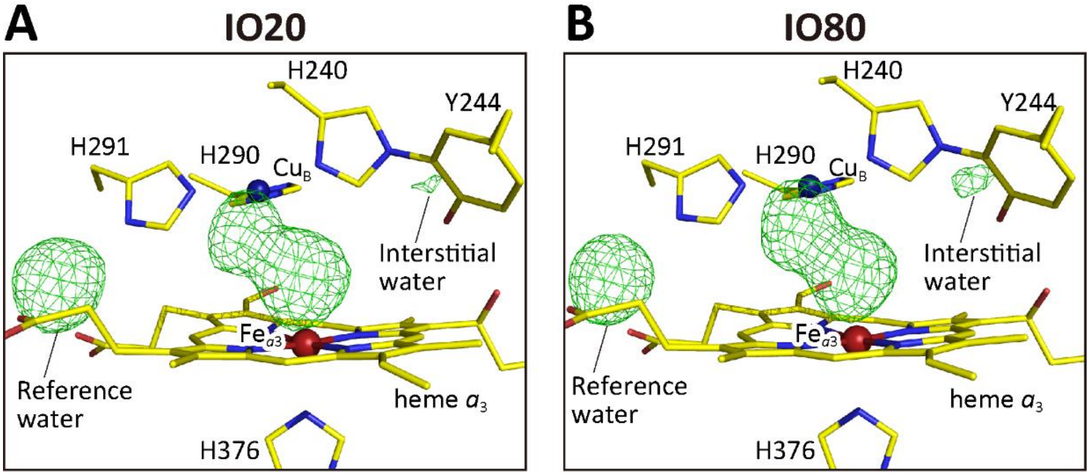

Figure S8. ***The interstitial waters in F_o-F_c maps of the O_2 reduction site of the X-ray structures of IO20 and IO80.*** *A* and *B*, F_o-F_c maps of the O_2 reduction site of IO20 and IO80 in monomer A at 1.80 Å resolution, given as *C* and *D* of Fig. 7 in the text, respectively, redrawn from the different angles for showing the elctron density cages of the interstitial waters, hydrogen-bonded to Tyr^{244}. The electron density cages were drawn at 3.0 σ.

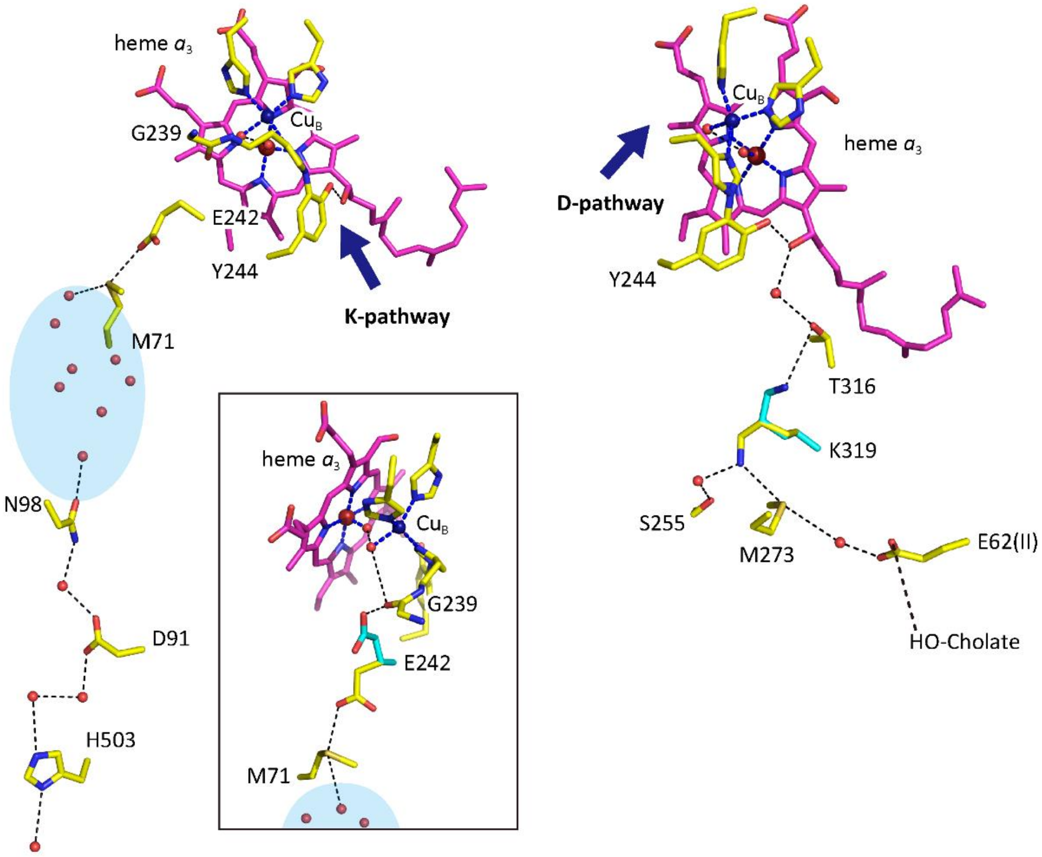

Figure S9. Possible structural changes in K and D-pathways, which could trigger proton transfers from the N-side to the O_2-reduction site. The red and dark blue spheres denote the locations of Fe$_{a3}$ and Cu$_B$. The small red spheres are placed at the sites of the fixed water or its derivatives (H_3O^+, OH^-, or O^{2-}) in the pathways. The dark blue arrows denote the locations of the O_2-reduction site-ends of the two pathways. The dotted lines are the hydrogen bonds in the pathways. The left figure shows the atomic model of the D-pathway with an inset showing the possible structural changes in Glu^{242} in light blue. The right figure includes possible structural changes in Lys^{319} in light blue. The atomic models without the structural changes were prepared from the X-ray diffraction data of PDB ID code 6JUW.

Table S1. Cu-O distances in four-coordination systems

States of Cu	Ligands	Cu-O distances (Å)	CSD ID	References
Cu^{1+} (X, Y, Z, OH^-)				
Cu^{1+}	OH^-	2.106	PENDUK	(42)
Cu^{1+}	OH^-	2.273	WACJAM	(43)
(Averaged Cu^{1+}-OH^- distance: 2.190Å)				
Cu^{1+} (X, Y, Z, H_2O)				
Cu^{1+}	H_2O	2.235	BIZBUH	(25)
Cu^{1+}	H_2O	2.262	WENNIO	(26)
Cu^{1+}	H_2O	2.105	DUBXUU	(27)
(Averaged Cu^{1+}-H_2O distance: 2.216Å)				
Cu^{2+} (N, N, N, OH^-)				
Cu^{2+}	OH^-	1.859	KAJLIT	(44)
Cu^{2+}	OH^-	1.848	UGAKL	(45)
(Averaged Cu^{2+}-OH^- distance: 1.854Å)				
Cu^{2+} (N, N, N, H_2O)				
Cu^{2+}	H_2O	1.926	UGALOG	(45)
Cu^{2+}	H_2O	1.958	OWEFED	(46)
(Averaged Cu^{2+}-H_2O distance: 1.942Å)				
Cu*	H_2O	1.971	OWEFED01	(46)
Cu*	H_2O	1.970	OWEFED01	(46)

*Electronic state of Cu is unknown.

Supporting Text 1

X-ray structural examination of flexibility of the critical residues of the substrate proton transfer pathways

Both the K- and D-pathway structures of bacterial and bovine CcOs indicate that, for proton transfers through the pathways, significant structural changes in at least one residue in each pathway are necessary as given in Fig. S9 (Glu^{242} in the D-pathway and Lys^{319} in the K-pathway). However, any significant X-ray structural difference in both pathways is not detectable among these intermediate forms determined in the present and previous papers (23). Therefore, we examined the influence of the changes in the oxidation and ligand binding state of the O_2-reduction site on the *B*-factors of these residues. For this purpose, the *B*-factor value of each critical residue was compared with the average *B*-factor value for the side chains of the helix including the concerned residue (residues 229–260 for Glu^{242} and residues 299–324 for Lys^{319}). The *B*-factors of Glu^{242} and Lys^{319} in the three intermediate forms (P, F, O, and E), together with those of the fully reduced and resting oxidized forms, were slightly higher (~10 %) and lower (~26%) than the average *B*-factors of the side chains of the helices including these residues, respectively. However, the *B*-factor difference values for each residues ((Glu^{242} and Lys^{319}) were identical with each other within the experimental errors. These X-ray structural findings strongly suggest that flexibility of these two residues is insensitive to the changes in the oxidation and ligand-binding sates of the O_2-reduction site as described in Fig. 1B. This finding indicates that these residues are activated only during the proton-pumping process and that spontaneous proton movements in these pathway are strictly controlled.

Supporting Text 2

Gating of the substrate proton transfer pathways

As described in Fig. 1B, the O_2 reduction site takes up four protons sequentially and alternatively. The Fergusson-Miller's group proposed an alternating access mechanism for the D and K substrate proton transfer pathways based on the following findings; disappearance of a water molecule (W301) hydrogen-bonded to Gly^{239} (in bovine number) in the upper end of the D-pathway in the X-ray structure of an oxidized bacterial CcO upon reduction of CcO (40) and redox-coupled X-ray structural changes in the relative location of Tyr^{244} and the OH group of the hydroxyfarnesyl ethyl group of heme a_3 in the upper end of the K-pathway (40).

However, in bovine CcO, the structure of the upper end of the K-pathway is redox-insensitive. The W301 is not detectable even in the composite omit maps of bovine CcO for P, F, O, E, the resting oxidized form, and the fully reduced form. These analyses suggest that the gating mechanism of bovine CcO is different from the one proposed for the bacterial CcO (40).

Supporting Text 3

A multiple structure of the hydroxyfarnesyl ethyl group of heme a

A multiple structure containing the oxidized- and reduced-type structures are detectable in the dimethylaryl group at the terminal of the hydroxyfarnesyl ethyl group of heme *a* in all the refined X-ray structures of the intermediate forms together with the resting oxidized (41) and fully reduced forms. This terminal dimethylaryl group forms the wall of the water channel of the H-pathway near the N-terminal entrance and thus the multiple structure may increase the flexibility of the water channel entrance to enhance the water exchange between the water channel and the N-side phase.

Supporting Text 4

Cu-O distances in tetra-coordinated copper complexes including OH^- or H_2O

A total of 11 copper complexes, each with four ligand atoms including an oxygen atom of OH^- or H_2O, collected from the Cambridge Crystallographic Structural Database (CSD) (42) show the Cu-O distances as listed in Table S1(25-27, 42-46). Four cupric complexes each with three nitrogen ligands and one oxygen ligand are given in the current CSD. The averaged Cu^{2+}-OH^- distance of 1.854 Å for the two complexes among the four complexes is significantly shorter than that of Cu^{2+}-H_2O of 1.942 Å for the other two complexes (Table S1). The comparison shows that influence of OH^-/H_2O difference on Cu^{2+}-O distance is detectable in the X-ray structures. No cuprous complex with four ligands consisting of three nitrogen atoms and one oxygen atom is given in the CSD. However, the CSD provides five cuprous complexes, each coordinated with one oxygen atom of OH^- or H_2O and three atoms different from oxygen (Table S1). In these cuprous complexes, the OH^-/H_2O structural difference does not significantly influence the Cu^{1+}-O distance. The Cu^{1+}-O distances of ~2.2 angstrom are significantly longer than those of Cu^{2+}-O.

References

40. Qin, L., Liu, J., Mills, D. A., Proshlyakov, D. A., Hiser, C., and Ferguson-Miller, S. (2009) Redox-dependent conformational changes in cytochrome *c* oxidase suggest a gating mechanism for proton uptake. *Biochemistry*. **48**, 5121–5130

41. Shinzawa-Itoh, K., Hatanaka, M., Fujita, K., Yano, N., Ogasawara, Y., Iwata, J., Yamashita, E., Tsukihara, T., Yoshikawa, S., and Muramoto, K. (2021) The 1.3-Å resolution structure of bovine cytochrome *c* oxidase suggests a dimerization mechanism. *BBA Advances* **1,** 100009.

42. Durini, S., Ardizzoia, G. A., Colombo, G., Therrien, B., and Brenna, S. (2018) H-bonding dependent phosphorescence in a mixed ligand copper(I) complex. *Polyhedron*. **139**, 189–195

43. Li, D., Li, R.-Z., Ni, Z., Qi, Z.-Y., Feng, X.-L., and Cai, J.-W. (2003) Synthesis and

crystal structure of photoluminescent copper(I)–phosphine complexes with oxygen and nitrogen donor ligands. *Inorg. Chem. Commun.* **6**, 469–473

44. Dhar, D., Yee, G. M., Spaeth, A. D., Boyce, D. W., Zhang, H., Dereli, B., Cramer, C. J., and Tolman, W. B. (2016) Perturbing the Copper(III)–Hydroxide Unit through Ligand Structural Variation. *J. Am. Chem. Soc.* **138**, 356–368

45. Dhar, D., Yee, G. M., and Tolman, W. B. (2018) Effects of Charged Ligand Substituents on the Properties of the Formally Copper(III)-Hydroxide ($[CuOH]^{2+}$) Unit. *Inorg. Chem.* **57**, 9794–9806

46. Bahnmüller, S., Plotzitzka, J., Baabe, D., Cordes, B., Menzel, D., Schartz, K., Schweyen, P., Wicht, R., and Bröring, M. (2016) Hexaethyltripyrrindione (H_3Et_6tpd): A Non-Innocent Ligand Forming Stable Radical Complexes with Divalent Transition-Metal Ions. *Eur. J. Inorg. Chem.* **2016**, 4761–4768

Movie S1.

Structure of the O_2 reduction site in each catalytic intermediate. This movie presents the X-ray structural changes in the ligand binding states of Fe_{a3} and Cu_B during the catalytic cycle composed of the six intermediate forms, R, OxyMb-type, P, F, O, and E. The OxyMb-type structure is given as a model of the A-form.
